# Solvent- and Catalyst-Free Environmentally Benign High Hydrostatic Pressure-Assisted Synthesis of Bioactive Hydrazones and the Evaluation of Their Stability Under Various Storage Conditions

**DOI:** 10.3390/molecules29225287

**Published:** 2024-11-08

**Authors:** Maximilian Costa, Frances Adhamidhi, Maxim Mastyugin, Adrianna R. Fusco, Alexander Lazarev, Zsuzsanna K. Zsengeller, Marianna Török, Béla Török

**Affiliations:** 1 Department of Chemistry, University of Massachusetts Boston, 100 Morrissey Blvd, Boston, MA 02125, USA; 2 Pressure BioSciences, Inc., Canton, MA 02375, USA; 3 Beth Israel Deaconess Medical Center, Boston, MA 02215, USA

**Keywords:** antioxidants, hydrazones, high hydrostatic pressure, pressure cycling, decomposition, antioxidant assays, DPPH, ABTS

## Abstract

Our group has seen great promise in using substituted diaryl-hydrazones to alleviate oxidative stress in preeclampsia. Specifically, fluorinated diaryl-hydrazones have shown great efficacy, confirmed via antioxidant assays and animal trials using pregnant mice. In addition to efficient antioxidant properties, these diaryl-hydrazones are also considered non-toxic. While the synthesis of these compounds is relatively simple, it commonly utilizes undesirable solvents and glacial acetic acid as the catalyst; additional solvents are needed for the isolation of the desired products, which negatively affects the green synthesis of the hydrazones. To combat this possible industrial roadblock, we have begun incorporating the use of hydrostatic high pressure (HHP) in the synthesis. The use of HHP allowed us to synthesize substituted diaryl-hydrazones in a 1:1 molar ratio without the need for solvents or acid catalysts. The optimized procedure can produce nearly quantitative yields, leading to an easier isolation of the products. Different HHP methodologies, such as constant high-pressure treatment and cycling (with different number of cycles, holding and decompression times) were applied and cycling was observed to be the most efficient activation for the majority of the reactions. Stability experiments were also conducted with one of the products and observed that although the solid-state storage does not alter the hydrazone, storing it in various solvents may significantly decrease the concentration of the active component which should be considered when performing the biochemical/biological assays.

## 1. Introduction

Preeclampsia is defined as a hypertensive disorder prevalent in up to 8% of all pregnancy-related complications across the globe, while being the cause of 16% of pregnancy-related deaths in higher-income countries and up to 26% of pregnancy-related deaths in lower-income countries, which calls for an increased focus in the development of new treatments on a global scale [1,2]. Relatively recent studies have highlighted a link between gestational hypoxia and the onset of preeclampsia for, as we know, gestational hypoxia leads to the formation of reactive oxygen species (ROSs), leading to increased oxidative stress in the placental tissue [3,4]. These findings point toward the utility of antioxidants in preeclampsia treatment [5,6,7]. Earlier we described highly effective diaryl-hydrazone-based antioxidants that showed promise as multifunctional drug candidates in Alzheimer’s disease exhibiting excellent anti-amyloid and radical scavenging activities [8,9]. Other reports also confirm the efficacy of hydrazones as radical scavengers [10,11,12,13]. In addition, these compounds are largely non-toxic and possess significant potential as drug candidates [14,15,16]. Recently, we have developed novel organofluorine hydrazones for mitochondrial antioxidant therapy to alleviate the symptoms of preeclampsia [17]. The above benefits of using hydrazones as drug candidates prompted us to re-evaluate the available synthetic protocols and develop a green methodology for their preparation.

Our attention turned primarily to the application of non-traditional activation methods that use alternative forms of energy [18]. One example, high hydrostatic pressure (HHP), utilizes mechanical compression to activate substrates [19,20]. The applicable high pressure ranges from 2 to 20 kbar and at the moment is mainly applied in the food industry [21]. While the first HHP-activated synthesis was reported in the 1970s [22], more recent examples in hydrogenation [23], Michael addition [24], Mannich reactions [25,26], lipase-catalytic esterification [27], nitro-aldol reaction [28], Michael [29], and aza-Michael [30,31], or Diels-Alder reactions [32,33], Friedel-Crafts alkylations [34], and the Paal-Knorr reaction [35] are also available.

Although due to the significance of hydrazones there are some green synthetic methods available for the preparation of these compounds [36,37,38,39,40,41,42], our primary goal was to develop a potentially solvent- and catalyst-free method by the application of high pressure. The major goal, in addition to developing such synthesis for the hydrazones, was to provide an illustration of the capabilities of high hydrostatic pressure-assisted synthesis. Extending our efforts in developing green synthetic protocols using non-traditional activation methods [43], herein we describe the HHP-assisted solvent- and catalyst-free preparation of bioactive diaryl-hydrazones as well as their chemical stability under various storage conditions.

## 2. Results and Discussion

**Scheme 1:**
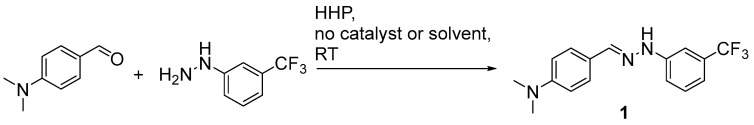
The general test reaction to evaluate the HHP-assisted catalyst- and solvent-free synthesis of diaryl-hydrazones.

The major aim of this work is to develop a new HHP-assisted solvent- and catalyst-free synthesis of diaryl-hydrazones, that have been found promising as drug candidates for mitochondrial antioxidant therapy in preeclampsia. Our earlier developed traditional synthesis, while effective, involves the use of dichloromethane (DCM) as a solvent, comparatively long reaction times, and energy intensive low (−20 °C) temperature [4]. Here, we explore the use of HHP under the target conditions, selecting one of the hydrazones that showed excellent activity in antioxidant biochemical assays, cell-based, and animal experiments as well. The general test reaction for evaluating the HHP-assisted synthesis method is illustrated in Scheme 1.

**Figure 1:**
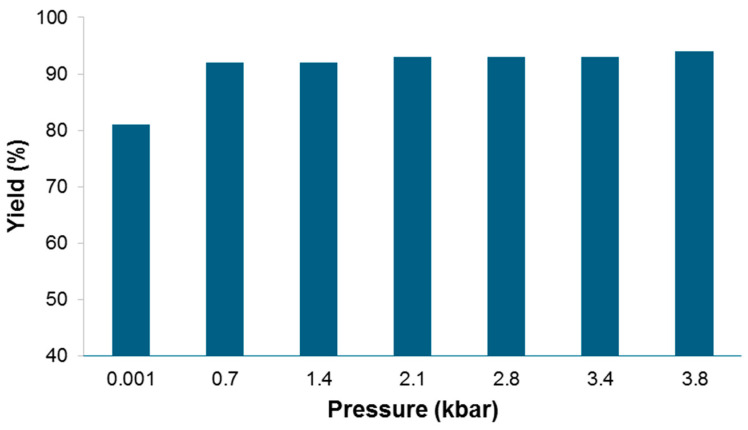
Effect of increasing hydrostatic pressure on the yield of the synthesis of (*E*)-N,N-dimethyl-4-((2-(3-(trifluoromethyl)phenyl)hydrazono)methyl)aniline (**1**) in a catalyst- and solvent-free reaction, at room temperature after 1 h reaction time using a 1:1 ratio (0.5 mmol each) of the starting materials.

First, the test reaction was carried out at different pressures to evaluate the effect increased pressure has on the product yield. The lowest pressure was the regular atmospheric pressure as a control, and it was gradually increased to 3.8 kbar. The plotted results are depicted in Figure 1.

Figure 1 shows that increasing pressure results in improved product yields. The reaction occurs at 1 bar pressure as well; however, applying 0.7 kbar pressure increased the yield by 11%. Further increases in the pressure to 1.4, 2.1, 2.8, 3.4, and finally 3.8 kbar resulted in small improvements in the product yields up to 94%. Essentially, the yield reached a plateau at 0.7–1.4 kbar and then remained the same. With this we could conclude that to reach the best yield at least 0.7 kbar is necessary and the 1.4 kbar pressure yield is firmly on the plateau which led to its selection for further investigations. However, it is also clear that further pressure increase is not necessary. The product was isolated from these reactions without any work up procedure and only a short air drying was applied to remove the byproduct water in those samples with quantitative conversion, while a short vacuum drying was required for the others. The elimination of purification, catalyst removal, and solvent handling is a notable advantage in green synthesis.

**Scheme 2:**
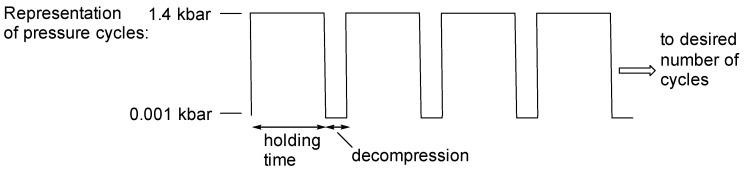
Schematic representation of the cycling experiments: the major variables are the applied pressure, the holding time, the length of decompression, and the number of cycles in the sequence.

With this data in hand, it has been decided to investigate the effect of different approaches of applying pressure on the reaction. The data exhibited in Figure 1 were determined under constant pressure. It means that the system was pressurized to the desired pressure and that pressure was maintained over the course of the reaction and released when the reaction time passed. Another, often more effective way of applying pressure is the so-called cycling, when compression-decompression cycles are applied in a predetermined sequence. In these investigations, 1.4 kbar was selected as applied pressure since it was on the steady part of the plateau for obtaining the maximum yield for the product in the previous constant pressure-assisted synthesis (Figure 1). The schematic representation of the cycling experiments is depicted in Scheme 2.

To begin these investigations, the pressure cycles were designed with the general depiction of the cycling experiments showing (Scheme 2) that the preset pressure was applied for a desired time which was followed by a decompression period also for a set time. Since the product yield plateaued at 1.4 kbar, in the first set of experiments the pressure was set to 1.4 kbar with 1 min holding time and 5 s decompression. In addition, several other options were tried for these variables, including longer or shorter holding times. The results are tabulated in Table 1.

The data show that the pressure cycling appears to be effective in increasing the reaction rates and providing higher yields at least under the optimized conditions as compared to the yields using constant pressure for much longer time (Figure 1). When the shortest cycle hold time was used (0.5 min), essentially it did not matter how many cycles were repeated; the yield was always around 80% with minimal increase when only two cycles were used (Table 1, entries 1–4). This essentially means that using just one cycle (i.e., 0.5 min pressure) resulted in 79% yield (Table 1, entry 4) about the same as the 1 h non-pressurized reaction (Figure 1). For clarity, we note that a single cycle is fundamentally the same as a constant pressure for that time. This was not the case when using 1 min holding time. The highest yield was obtained when the number of cycles was ten (Table 1, entry 5), which gradually declined when the number of cycles were decreased to 5, 2, and 1 (Table 1, entries 6–8). The highest yield (96%, 10 × 1 min cycles) exceeds the yield that was obtained by the constant 1 h pressure treatment despite the significantly decreased overall time (1 h vs. 10 min) indicating the positive effect that the cycling provides. The lowest yield was similar to those obtained with the 0.5 min holding time. Further increasing the holding time to 2 min in each cycle increased the average yield across the measurements to about 85%, however the best yield was somewhat lower that that obtained with the 1 min holding time (93% vs. 96%, Table 1, entries 10 and 5). Based on these experiments, it can be concluded that cycling appears to improve the yield, even when the overall time spent under pressure is only 10 min. Therefore, it has been decided to explore the scope of the HHP-assisted reaction by using 10 × 1 min cycles with several benzaldehydes and phenylhydrazines to prepare the hydrazones. However, it must be noted that these experiments have been carried out multiple times (3–4 individually) and the data obtained in the 10 × 2 min cycles are statistically too close to that of the 10 × 1 min setup to make a clear selection based on yield alone. Thus, since the 10 × 1 min overall takes half the time of the other setup, it has been chosen for the evaluation of the scope. The data are tabulated in Table 2.

Table 2 describes that all the reactions occurred readily in a catalyst- and solvent-free environment. It is also shown that to a varying degree, the reactions generally produce higher yields under pressurized conditions. In some cases, the HHP reactions resulted in nearly quantitative yields upon the HHP cycling (Table 2, entries 2, 4) producing 10–20% higher yield than the control experiments. There are several other examples when the HHP-assisted reaction provided good yields (> or ~80%) and about 10% improvement. In two examples, the HHP reactions resulted in a 30–40% increase in product yields (Table 2, entries 7 and 8). In this case the control reaction showed a low 37% yield only. Finally, there was one case when the reactions were both excellent in the 10 min reaction time reaching 98% yields with no difference between the methods (Table 2, entry 3). In addition, in three cases, the 1 h constant pressure provided better yields; hence we reported those in Table 2 (entries 2, 8, 9) and the respective yields with cycling were about 10% lower in each case. As a partial summary it could be noted that although the use of pressure often results in considerable improvement in yield, every reaction is unique and should be evaluated individually.

**Figure 2:**
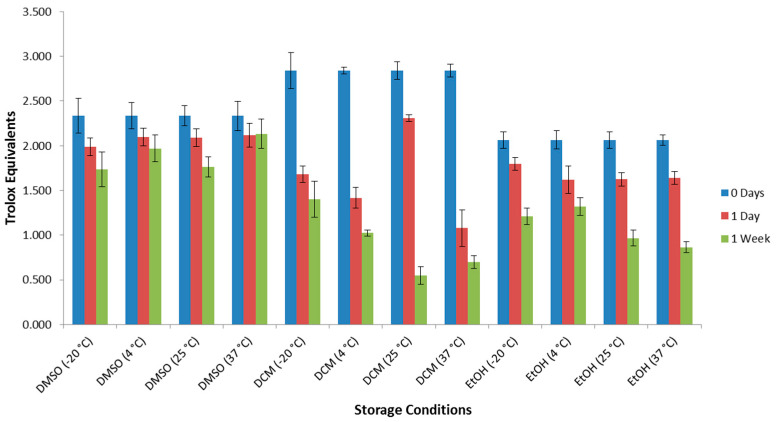
Monitoring the deterioration of the antioxidant effect of compound **3e** upon storage under different conditions, including the solvent and temperature by the DPPH assay. All samples with **3e** the concentration of 10 mM were kept in the dark throughout the storage.

**Figure 3:**
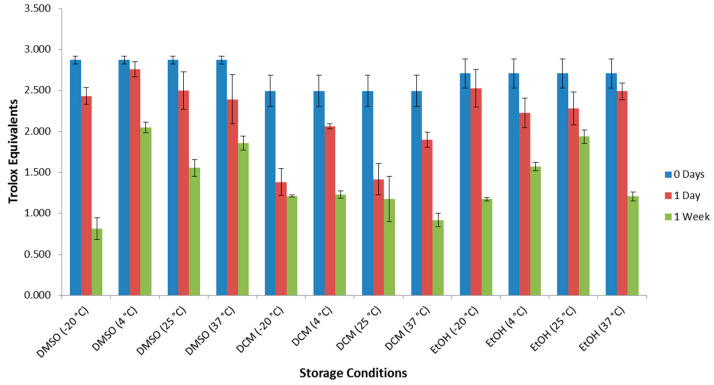
Monitoring the deterioration of the antioxidant effect of compound **3e** upon storage under different conditions, including the solvent and temperature by the ABTS assay. All samples with **3e** concentration of 50 mM were kept in the dark throughout the storage.

After evaluating the effect of HHP on the synthesis of the antioxidant hydrazones, we have selected the most active compound (**1**) that has the greatest likelihood of advancing to the next level of the project: the animal studies. The stability of **1** was evaluated under various conditions, including storage in different solvents at different temperatures, all under regular laboratory atmosphere. It must be noted, however, that this compound was first synthesized in our laboratory over a decade ago as a potential anti-Alzheimer’s disease agent [8]. It has been stored in solid, crystalline form and even after 12 years of storage, it did not show any deterioration in its purity. Thus, we can conclude that the shelf-life of this compound is outstanding and can be measured in several years, which is a promising feature for a drug candidate. However, when assays are carried out, the compounds are dissolved and used in a solution form and thus it is important to determine the time and conditions in which these dissolved samples can be used. The stability of the **3e** samples was observed by GC-QTOF analysis as well as determining the remaining antioxidant activity of the compound. For these measurements, the commonly applied 2,2-diphenyl-picryl-hydrazyl (DPPH), and 2,2′-azino- bis(3-ethylbenzothiazoline-6-sulphonic acid) (ABTS) assays were used [5,8]. The results of the antioxidant assays are depicted in Figure 2 and Figure 3.

Summarizing the individual results, Table 3 shows the percentile deterioration of the antioxidant activity of **3e** in the DPPH, the ABTS assay, and the analytical characterization of the sample after one week of storage.

**Figure 4:**
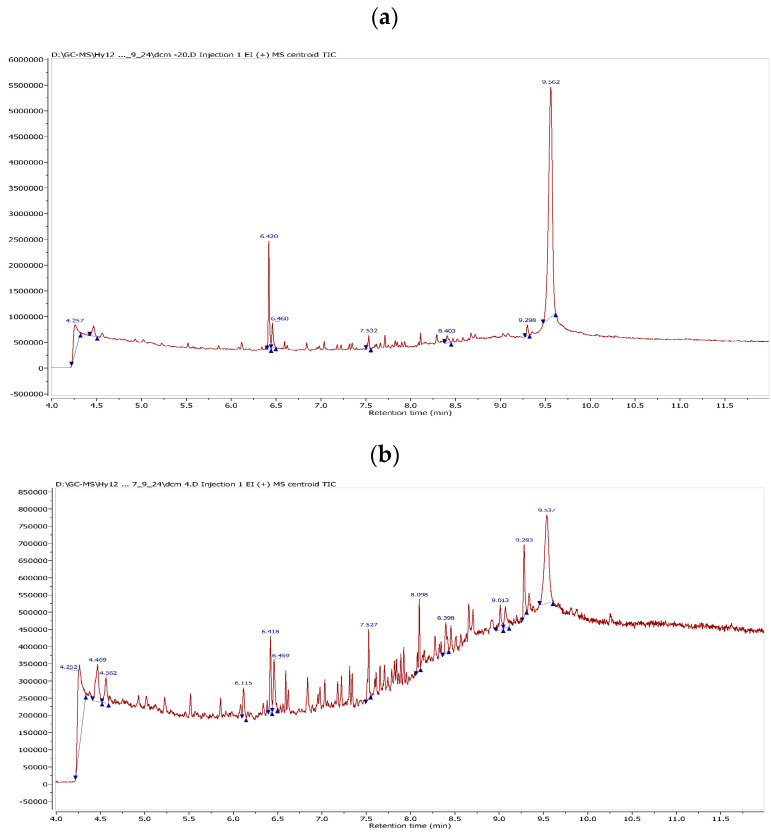
Monitoring the deterioration of the concentration of compound **3e** upon storage in (**a**) DCM at −20 °C, and (**b**) DCM at 4 °C. The peaks at 4.2 min and 6.4 min are the nominal hydrolysis products.

The data in Figure 2 and Figure 3 unambiguously show that the antioxidant activity of **1** decreases over time as evidenced by both assays. The deterioration of the radical scavenging activity is relatively slow under some conditions (Table 3, entries 2–4, 11) and quite rapid under others (Table 3, entries 6–8). Overall, the DPPH assay detected higher remaining activity than the ABTS assay did, although there are conflicting data pairs. Dichloromethane (DCM) appears to be the worst solvent in terms of losing antioxidant potential, in contrast to dimethylsulfoxide (DMSO) that ensured some of the highest remaining activities. While the GC-MS data supported the decline of **1** concentration after the storage in all solvents, the numerical values show some discrepancy when compared to the remaining antioxidant activity. This can be partially explained by the analysis of the product mixtures after decomposition. The typical total ion chromatogram of a relatively clean decomposition is shown as an example in Figure 4a, and a largely uncontrolled multicomponent mixture in Figure 4b.

Regardless of the type of mixture, it appears that the expected hydrolysis products can be found in every sample. The 3-trifluoromethyl-phenylhydrazine (4.2 min retention time) and the 4-dimethylaminobenzaldehyde (6.4 min retention time) can be found in every sample indicating that the hydrolysis is the major route of decomposition. It is highly prevalent in DCM where the inherent acidity of the solvent can catalyze the hydrolysis in the presence of moisture. The presence of 3-trifluoromethyl-phenylhydrazine in the decomposition mixtures also partially explains why the actual antioxidant activity may be higher than the expected value based on the analytical data. The hydrazines themselves are active antioxidants and hence their presence and activity accounts for the often-misleading differences in the antioxidant and analytical data. However, there could be other decomposition products that possess radical scavenging activity.

The decomposition data clearly confirm that **1**, while showing superior stability in its crystalline form, should be handled with care after it is dissolved in essentially any solvents. When the compound is used in an assay, it is best to prepare the solutions fresh, and not use premade and stored samples, due to the considerable decrease in its concentration after several days of storage.

## 3. Materials and Methods

### 3.1. General Information

All substrates and solvents were purchased from Sigma Aldrich (St. Louis, MO, USA) and used without further purification.

The ^1^H and ^13^C NMR and ^19^F spectra were obtained on 400 MHz Agilent MM2 NMR spectrometers (Agilent, Santa Clara, CA, USA), in DMSO-*d_6_* with using the signal of either tetramethylsilane or the residual solvent signal as a reference. The temperature was 25 °C (accuracy ± 1 °C). The mass spectrometric identification and purity determination of the products have been carried out by an Agilent 6850 gas chromatograph-5973 mass spectrometer system (70 eV electron impact ionization) using a 30 m long DB-5 type column (Agilent, Santa Clara, CA, USA). Additional analysis and the HRMS data determination were carried out using an Agilent 7250 GC-QTOF mass spectrometer operated in electron impact ionization (EI, 70 eV) mode.

### 3.2. General Synthesis of the Hydrazones Under High Hydrostatic Pressure

A mixture of 1:1 molar ratio of phenylhydrazine (0.5 mmol) and benzaldehyde (0.5 mmol) were placed into a 150 μL high-pressure reaction tube. The reaction tubes were sealed using PCT microcaps. One of the reaction tubes was placed in the chamber compartment of the barocycler 2320EXT (Pressure BioSciences, Inc., Canton, MA, USA), while the other was left on the bench top. Each hydrazone was synthesized at the desired pressure and method within the barocycler. After the reaction the products were isolated as solids of various colors and were purified by recrystallization in 95% aq. EtOH. Then the product was dissolved in ethyl acetate and the yield and purity of the product was analyzed by the gas-chromatography/time-of-flight mass spectrometry (GC-TOFMS) (Agilent, Santa Clara, CA, USA). This process was repeated using varying time intervals with constant temperature and pressure to optimize the yield and time. The cycling mode of the barocycler was also applied and was found to be an effective method in synthesizing the products, by using a predetermined number of cycles, with holding time and decompression. The reactions have been carried out several times, thus the reported numbers are averages of at least three experiments.

### 3.3. Preparation of Samples for Decomposition Tests by Antioxidant Assays

The **1** samples were weighed and dissolved in the solvent in either a 1.5 mL Eppendorf tube (ThermoFisher Sci., Waltham, MA, USA) (DMSO or EtOH) or a 2 dram amber screw cap vial (DCM) to a concentration of 50 mM. For the ABTS stock solutions with DMSO and EtOH, 60 μL of the 50 mM solution was pipetted into three Eppendorf tubes for each solvent, totaling four Eppendorf tubes per solvent. Each of the Eppendorf tubes were stored in their respective incubation temperatures (−20 °C, 4 °C, 25 °C, or 37 °C) in the dark. For the ABTS stock solutions of DCM, 1 mL of the solution was pipetted into two amber screw cap vials and one 2 mL amber crimp cap autosampler vial. Each of the screw cap vials and the autosampler vial were stored in their respective incubation temperatures (−20 °C, 4 °C, or 25 °C for the screw cap vials and 37 °C for the autosampler vial) in the dark. To create the 10 mM DPPH stock solutions, 25 μL of the 50 mM ABTS stock solution was added to 100 μL of the respective solvent in a 1.5 mL Eppendorf tube with those containing DCM being covered with parafilm. The Eppendorf tubes were stored in their respective incubation temperatures in the dark.

### 3.4. Preparation of Samples for Decomposition Tests by GC-QTOFMS Analysis

The **1** samples (0.5 mL, 1 μg/mL) were placed in twelve 2.0 mL GC-MS autosampler vials. The vials were labeled based on solvent added, and conditions they were placed in. The solvents utilized were dichloromethane (DCM), dimethyl sulfoxide (DMSO), and 75 mM ethanol (EtOH), respectively. The samples were left in each condition for 1 week before separation and analysis.

### 3.5. Separation of Sample from DMSO

DMSO being soluble within DCM required multiple extractions using lithium chloride (LiCl) (5% *w*/*v*, an equivalent volume of LiCl solution was added to the DMSO solution and shaken well to be extracted by DCM). LiCl was used to interact with DMSO to separate it from the more polar hydrazone compound. The DCM was then used as the organic layer in the solution to extract the hydrazone from the DMSO. The DMSO sample and equal volume (1.5 mL) of LiCl was added to the test tube and vortexed. DCM was then added to the test tube. The mixture was vortexed until combined. The organic layer was collected and placed into another labeled test tube. This procedure was performed 5 times for each sample.

### 3.6. Gas Chromatography Mass Spectroscopy of the Samples

Samples were diluted with 10 mL of DCM into labeled test tubes. Into each labeled autosampler vial, 2–3 drops of the diluted solution were added and filled to 1.5 mL with DCM. Samples were tested by gas-chromatography/time-of-flight mass spectrometry (GC-TOFMS).

### 3.7. DPPH Radical Scavenging Assay of the Samples

The DPPH assay was conducted following earlier procedures [9]. A VersaMax UV-Vis plate reader was set to 519 nm and 37 °C and used with the SoftMax Pro 5 software (Molecular Devices, San Jose, CA, USA) in order to assess the scavenging of the DPPH radical by the hydrazones. For more details, see our earlier publications [9]. The data were processed using the equation below where Abs_c_ is the absorbance of the control and Abs_t_ is the absorbance of the test sample.

Percent Radical Scavenging = ((〖Abs〗_c_ − 〖Abs〗_t_))/〖Abs〗_c_ × 100

### 3.8. ABTS Radical Scavenging Assay of the Samples

Following our earlier procedure [9], the ABTS assay was carried out to determine the radical scavenging activity of these hydrazones. The ABTS radical was generated 12 to 24 h before running the assay by dissolving ABTS to the concentration of 7 mM, and K_2_S_2_O_8_ to the concentration of 2.45 mM, together in 4 mL of DI water. A VersaMax UV-Vis plate reader was set to 734 nm and 37 °C and used with the SoftMax Pro 5 software (Molecular Devices) in order to assess the scavenging of the ABTS radical by the compounds investigated. For more details, see our earlier publications [9]. The data were processed using the equation below where Abs_c_ is the absorbance of the control and Abs_t_ is the absorbance of the test sample.

Percent Radical Scavenging = ((〖Abs〗_c_ − 〖Abs〗_t_))/〖Abs〗_c_ × 100

The structural identification of the hydrazones was carried out using ^1^H, ^13^C and ^19^F (when applicable) NMR spectroscopy and high-resolution mass spectrometry (HR-MS). All HR-MS data are within the 5 ppm limit for the difference as compared to the calculated values. The spectra can be found in the Supplementary Materials File S1.

**(*E*)-N,N-dimethyl-4-((2-(3-(trifluoromethyl)phenyl)hydrazono)methyl)aniline (1)** (refs. [8,12])
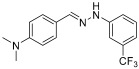
**^1^H NMR** (400 MHz, DMSO-*d_6_*) δ (ppm): 10.32 (s, 1H, NH), 7.85 (s, 1H, CH=), 7.51 (d, 2H, *J* = 8.0 Hz), 7.39 (t, 1H, *J* = 7.8 Hz), 7.34 (s, 1H), 7.27 (d, 1H, *J* = 7.9 Hz), 6.99 (d, 1H, *J* = 7.8 Hz), 6.71 (d, 2H, *J* = 4.1 Hz), 2.91 (s, 6H).
**^13^C NMR** (100.58 MHz, DMSO-*d_6_*) δ (ppm): 151.2, 147.1, 140.2, 130.7 (q, C-CF_3_, *J* = 31 Hz), 130.6, 127.8, 125.2 (q, CF_3_, *J* = 273 Hz), 123.7, 115.9, 114.4, 112.6, 108.1 (q, C-C-CF_3_, *J* = 4 Hz), 41.3
^19^F NMR (376 MHz, DMSO-*d_6_*) δ (ppm): −61.55
**HRMS** C_16_H_16_F_3_N_3_: Calc.: 307.12963 Found: 307.12853

**(*E*)-1-benzylidene-2-phenylhydrazine (2)** (ref. [8])

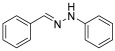

**^1^H NMR** (400 MHz, DMSO-*d_6_*), δ (ppm): 10.33 (s, 1H, NH), 7.86 (s, 1H, CH=), 7.63 (d, 2H, *J* = 4.1 Hz), 7.36 (t, 2H, *J* = 7.2 Hz), 7.27–7.19 (m, 3H), 7.08 (d, 2H, *J* = 4.0 Hz) 6.73 (t, 1H, *J* = 7.8 Hz)
**^13^C NMR** (100.58 MHz, DMSO-*d_6_*) δ (ppm): 145.7, 136.8, 136.3, 129.6, 129.1, 128.3, 126.1, 119. 2, 112.4.
**HRMS** C_13_H_12_N_2_: Calc.: 196.10005, Found: 196.09898

**(*E*)-1-benzylidene-2-(3-(trifluoromethyl)-phenylhydrazine (3)** (ref. [12])

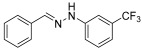

**^1^H NMR** (400 MHz, DMSO-*d_6_*) δ 10.68 (s, 1H, NH), 7.93 (s, 1H, CH=), 7.66 (d, 2H, *J* = 4.1 Hz), 7.41–7.25 (m, 6H), 7.02 (d, 1H, *J* = 4.5 Hz).
**^13^C NMR** (100.58 MHz, DMSO-*d_6_*) δ 146.4, 138.7, 135.8, 130.5 (q, C-CF_3_, *J* = 30 Hz), 130.4, 129.0, 128.8, 126.3, 124.9 (q, CF_3_, *J* = 272 Hz), 116.0, 115.1, 108.3.
**^19^F NMR** (376 MHz, DMSO-*d_6_*) δ (ppm): −61.6
**HRMS** C_14_H_11_F_3_N_2_: Calc.: 264.08743, Found: 264.09200

**(*E*)-1-benzylidene-2-(1,2,3,4,5-pentafluoro)-phenylhydrazine (4)**

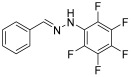

**^1^H NMR** (400 MHz, DMSO-*d_6_*) δ 10.28 (s, 1H, NH), 8.08 (s, 1H, CH=), 7.56 (d, 2H, *J* = 4.1 Hz), 7.36 (t, 2H, *J* = 8 Hz), 7.30 (t, 1H, *J* = 7.9 Hz).
**^13^C NMR** (100.58 MHz, DMSO-*d_6_*), δ 142.4, 139.3 (m), 136.7 (m), 135.2, 132.9 (m), 129.3, 129.1, 126.4, 121.7.
**^19^F NMR** (376 MHz, DMSO-*d_6_*) δ (ppm): −156.0, −164.5, −170.3
**HRMS** C_13_H_7_F_5_N_2_: Calc.: 286.05294; Found: 286.04846

**(*E*)-N,N-dimethyl-4-((2-phenyl)hydrazynylidene)methyl)aniline (5)**

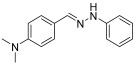

**^1^H NMR** (400 MHz, DMSO-*d_6_*) δ 9.04 (s, 1H, NH), 6.87 (s, 1H, CH=), 6.55 (d, 2H, *J* = 3.9 Hz), 6.27(t, 2H, *J* = 7.5 Hz), 6.13 (d, 2H, *J* = 4.0 Hz), 5.78 (m, 3H), 1.98 (s, 6H, CH_3_).
**^13^C NMR** (100.58 MHz, DMSO-*d_6_*), δ 149.8, 145.4, 137.3, 126.7, 126.4, 123.2 117.4, 111.6, 111.2, 39.4.
**HRMS** C_15_H_17_N_3_: Calc.: 239.14225 Found: 239.14263

**(*E*)-4-((2-phenylhydrazono)methyl)phenol (6)** (ref. [8])

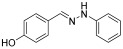

**^1^H NMR** (400 MHz, DMSO-*d_6_*), δ (ppm) 9.98 (s, 1H, NH), 9.61 (s, broad, 1H, OH), 7.76 (s, 1H, CH=), 7.45 (d, 1H, *J* = 4.0 Hz), 7.16 (t, 3H, *J* = 4.1 Hz), 7.00 (d, 3H, *J* = 3.9 Hz), 6.78 (d, 1H, *J* = 4.2 Hz), 6.66 (t, 1H, *J* = 4.1 Hz).
**^13^C NMR** (100.58 MHz, DMSO-*d_6_*), δ (ppm) 158.1, 146.1, 137.5, 129.4, 127.6, 127.4, 118.5, 115.9, 112.1.
**HRMS** C_13_H_12_N_2_O: Calc.: 212.09469; Found: 212.10139

**(*E*)-1-(4-hydroxybenzylidene)-2-(3-(trifluoromethyl)-phenylhydrazine (7)**

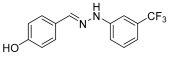

**^1^H NMR** (400 MHz, DMSO-*d_6_*) δ 10.39 (s, 1H, NH), 9.69 (s, 1H, OH), 7.85 (s, 1H, CH=), 7.51 (d, 2H, *J* = 4 Hz), 7.36 (t, 1H, *J* = 8.0 Hz), 7.31 (s, 1H), 7.24 (d, 1H, *J* = 3.9 Hz), 6.97 (d, 1H, *J* = 4.1 Hz), 6.82 (d, 2H, *J* = 8.3 Hz).
**^13^C NMR** (100.58 MHz, DMSO-*d_6_*), δ 158.6, 146.7, 139.4, 130.4 (q, C-CF_3_, *J* = 30 Hz), 130.4, 128.0, 124.9 (q, CF_3_, *J* = 271 Hz), 126.8, 116.1, 115.8, 114.4, 107.9.
**^19^F NMR** (376 MHz, DMSO-*d_6_*) δ (ppm): –61.6.
**HRMS** C_14_H_11_F_3_N_2_O: Calc.: 280.08235; Found: 280.08752

**(*E*)-1-(3,4-dihydroxybenzylidene)-2-phenylhydrazine (8)**

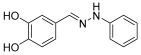

**^1^H NMR** (400 MHz, DMSO-*d_6_*) δ 9.07 (s, 1H, NH), 8.93 (s, 2H, OH), 7.73 (s, 1H, CH=), 7.21–7.19 (s, 1H), 7.14 (m, 4H), 7.12 (d, 1H, *J* = 8.2 Hz), 6.97 (t, 1H, *J* = 8.0 Hz), 6.76 (t, 1H, *J* = 7.9 Hz)
**^13^C NMR** (100.58 MHz, DMSO-*d_6_*) δ 146.6, 146.2, 146.0, 137.9, 129.5, 127.8, 118.9, 118.5, 116.0, 112.3, 112.1.
**HRMS** C_13_H_10_N_2_O_2_: Calc.: 226.07423; Found: 226.07351

**(*E*)-1-(3,4-dimethoxybenzylidene)-2-phenylhydrazine (9)** (ref. [8])

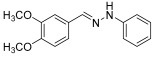

**^1^H NMR** (400 MHz, DMSO-*d_6_*), δ (ppm) 10.15 (s, 1H, NH), 7.80 (s, 1H, CH=), 7.30 (s, 1H), 7.19 (t, 1H, *J* = 4.1 Hz), 7.06 (d, 3H, *J* = 4 Hz), 6.92 (d, 2H, *J* = 4 Hz), 6.70 (t, 1H, *J* = 8.2 Hz), 3.79 (s, 3H), 3.74 (s, 3H).
**^13^C NMR** (100.58 MHz, DMSO-*d_6_*), δ (ppm) 149.6, 149.5, 146.0, 137.2, 129.5, 129.2, 119.9, 118.8, 112.3, 112.0, 108.1, 56.3, 55.9.
**HRMS** C_15_H_16_N_2_O_2_: Calc.: 256.12118 Found: 256.12112

## 4. Conclusions

In conclusion, high hydrostatic pressure (HHP) was found to be an efficient activation method for the synthesis of hydrazones from substituted benzaldehydes and phenylhydrazines. The HHP-led approach has several benefits including the solvent and catalyst-free reaction conditions, thus eliminating catalyst/excess reagent disposal. In addition, the reactions occur at room temperature, with ~90% atom economy with high to excellent yields and a simple recrystallization in 95% aq. EtOH is required for purification. The HHP instrument allowed for safe and easy-to-carry-out protocols that are tunable in terms of pressure, temperature, reaction time, or pressure cycles. It was found that the pressure cycling mode was the most efficient in this reaction, often offering nearly quantitative yields after ten 1 min cycles.

The stability measurements indicated that the compounds showed exceptional stability in their solid, crystalline form; however, they also showed considerable decomposition in all solvents used for storage as stock solutions, which was observed by assessing the remaining antioxidant activity in assays, as well as by gas chromatography mass spectrometric analysis. These data highlight that the compounds are better prepared freshly before any assays.

## Figures and Tables

**Table 1:** Optimization of the pressure cycling for the synthesis of **3e** ^a^.

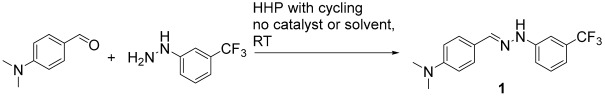					
Entry	P (kbar)	Holding Time (min)	Decompression Time (s)	Number of Cycles	Yield ^b^ (%)
1	1.4	0.5	5	10	79
2	1.4	0.5	5	5	81
3	1.4	0.5	5	2	83
4	1.4	0.5	5	1	79
5	1.4	1	5	10	96
6	1.4	1	5	5	86
7	1.4	1	5	2	79
8	1.4	1	5	1	75
9	1.4	2	5	10	88
10	1.4	2	5	5	93
11	1.4	2	5	2	87
12	1.4	2	5	1	80

^a^ Reaction conditions: 0.5 mmol of aldehyde, 0.5 mmol of hydrazine, neat mixture, ^b^ Determined by GC-MS.

**Table 2:** Ambient temperature synthesis of hydrazones by a HHP-assisted solvent- and catalyst-free protocol ^a^.

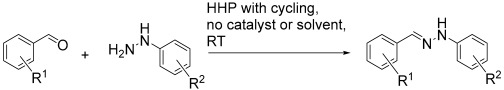						
Entry	R^1^	R^2^	Product	Time (min)	HHP Yield ^b^ (%)	Control Yield ^c^ (%)
1	4-N(CH_3_)_2_	3-CF_3_	**1**	10	96	75
2 ^d^	H	H	**2**	60	96	86
3	H	3-CF_3_	**3**	10	98	98
4	H	2,3,4,5,6-F	**4**	10	96	77
5	4-N(CH_3_)_2_	H	**5**	10	77	70
6	4-OH	H	**6**	10	75	64
7	4-OH	3-CF_3_	**7**	10	77	37
8 ^d^	3,4-(OH)_2_	H	**8**	60	54	24
9 ^d^	3,4-(OCH_3_)_2_	H	**9**	60	76	70

^a^ Reaction conditions: 0.6 mmol of **1**, 0.6 mmol of **2**, neat mixture; ^b^ isolated yields using HHP cycling, 10 cycles of 1 min holding with 5 s decompression; ^c^ isolated yields using ambient 1 bar pressure; ^d^ 1 h constant pressure.

**Table 3:** The remaining antioxidant activity after 7 days of storage was determined by the DPPH and ABTS assays, as well as the decomposition of **1** by GC-QTOF MS analysis ^a^.

Entry	Storage Conditions ^b^	Remaining Antioxidant Activity by DPPH Assay (%) ^c^	Remaining Antioxidant Activity by ABTS Assay (%) ^d^	1 Concentration by GC-QTOF MS Analysis (%) ^e^	Notes
1	DMSO/−20 °C	74	28	18	multiple products
2	DMSO/4 °C	84	71	8	multiple products
3	DMSO/25 °C	76	54	8	multiple products
4	DMSO/37 °C	92	65	13	multiple products
5	DCM/−20 °C	49	49	72	hydrolysis products
6	DCM/4 °C	36	49	25	hydrolysis and many related products
7	DCM/25 °C	19	47	64	mainly hydrolysis products
8	DCM/37 °C	25	37	47	hydrolysis products dominate, with many small peaks
9	EtOH/−20 °C	59	43	12	multiple products
10	EtOH/4 °C	64	58	7	hydrolysis products dominate, with many small peaks
11	EtOH/25 °C	47	72	7	hydrolysis products dominate, with many small peaks
12	EtOH/37 °C	42	45	15	multiple products

^a^ Compared to the activity at start (100%); ^b^ DMSO-dimethylsulfoxide, DCM-dichloromethane, EtOH-ethanol; ^c^ 10 mM stock solution was used for DPPH assay; ^d^ 50 mM stock solution was used for ABTS assay; ^e^ 100 mM stock solution was used for the GC-MS analysis.

## Data Availability

The original contributions presented in the study are included in the article/Supplementary Material, further inquiries can be directed to the corresponding author/s.

## Supplementary Materials

The following supporting information can be downloaded at: https://www.mdpi.com/article/10.3390/molecules29225287/s1.
