# Supplementary material for: Solvent- and Catalyst-Free Environmentally Benign High Hydrostatic Pressure-Assisted Synthesis of Bioactive Hydrazones and the Evaluation of Their Stability Under Various Storage Conditions
Source: Molecules. 2024 Nov 8;29(22):5287. doi: 10.3390/molecules29225287 (PMC11596250; doi:10.3390/molecules29225287)
Supplement: Supplementary file 1 [file molecules-29-05287-s001.zip › molecules-3174688-supplementary.pdf]

SUPPORTING INFORMATION

to

Solvent- and catalyst-free environmentally benign high hydro-static pressure-assisted synthesis of bioactive hydrazones and the evaluation of their stability under various storage conditions

by Maximilian Costa,[1] Frances Adhamidhi,[1] Maxim Mastyugin,[1] Adrianna R. Fusco,[1] Alexander Lazarev,[2] Zsuzsanna K. Zsengeller,[3] Marianna Török,[1]* Béla Török,[1]*

[1] *Department of Chemistry, University of Massachusetts Boston, 100 Morrissey Blvd, Boston, MA, USA email: Bela.Torok@umb.edu*

[2] *Pressure BioSciences, Inc., Canton, MA 02375, USA*

[3] *Beth Israel Deaconess Medical Center, Boston, MA 02215, USA*

1. General information

General information. All substrates and solvents were purchased from Sigma Aldrich and used without further purification.

The ^{1}H and ^{13}C NMR and ^{19}F spectra were obtained on a 300 MHz Varian and a 400 MHz Agilent MM2 NMR spectrometers, in $CDCl_3$ or DMSO-d_6 with using the signal of either tetramethylsilane or the residual solvent signal as a reference. The temperature was 25 °C (accuracy ±1 °C). The mass spectrometric identification and purity determination of the products have been carried out by an Agilent 6850 gas chromatograph-5973 mass spectrometer system (70 eV electron impact ionization) using a 30m long DB-5 type column (J&W Scientific). Additional analysis and the HRMS data determination were carried out using an Agilent 7250 GC-QTOF mass spectrometer operated in electron impact ionization (EI, 70 eV) mode.

General synthesis of the hydrazones under high hydrostatic pressure

Into a 150 µL high-pressure reaction tube, a mixture of 1:1 molar ratio of phenylhydrazine (0.5 mmol) and benzaldehyde (0.5 mmol) were placed. The reaction tubes were sealed using PCT microcaps. One of the reaction tubes was placed in the chamber compartment of the Barocycler 2320EXT (Pressure BioSciences, Inc.), while the other was left on the bench top. Each hydrazone was synthesized at the desired pressure and method within the barocycler. After the reaction the product was isolated as solids of various colors and were purified by recrystallization in 95% aq. EtOH. Then the product was dissolved in ethyl acetate and the yield

and purity of the product was analyzed by the gas-chromatography/time-of-flight mass spectroscopy (GC-TOFMS). This process was repeated using varying time intervals with constant temperature and pressure to optimize the yield and time. The cycling mode of the Barocycler was also applied and was found to be an effective method in synthesizing the products, by using a predetermined number of cycles, with holding time and decompression.

Preparation of Samples for Decomposition Tests by Antioxidant Assays

The 1 samples were weighed and dissolved in the solvent in either a 1.5 mL Eppendorf tube (DMSO or EtOH) or a 2 dram amber screw cap vial (DCM) to a concentration of 50 mM. For the ABTS stock solutions with DMSO and EtOH, 60 µL of the 50 mM solution was pipetted into three Eppendorf tubes for each solvent, totaling four Eppendorf tubes per solvent. Each of the Eppendorf tubes were stored in their respective incubation temperatures (-20 °C, 4 °C, 25 °C, or 37 °C) in the dark. For the ABTS stock solutions of DCM, 1 mL of the solution was pipetted into two amber screw cap vials and one 2 mL amber crimp cap autosampler vial. Each of the screw cap vials and the autosampler vial were stored in their respective incubation temperatures (-20 °C, 4 °C, or 25 °C for the screw cap vials and 37 °C for the autosampler vial) in the dark. To create the 10 mM DPPH stock solutions, 25 µL of the 50 mM ABTS stock solution was added to 100 µL of the respective solvent in a 1.5 mL Eppendorf tube with those containing DCM being covered with parafilm. The Eppendorf tubes were stored in their respective incubation temperatures in the dark.

Preparation of Samples for Decomposition Tests by GC-QTOFMS Analysis

The 1 samples (0.5 mL, 1 µg/mL) were placed in twelve 2.0 mL GC-MS autosampler vials. The vials were labeled based on solvent added, and conditions they were placed in. The solvents utilized were dichloromethane (DCM), dimethyl sulfoxide (DMSO), and 75 mM ethanol (EtOH), respectively. The samples were left in each condition for 1 week before separation and analysis

Separation of Sample from DMSO

DMSO being soluble within DCM required multiple extractions using lithium chloride (LiCl) (5 % w/v, an equivalent volume of LiCl solution was added to the DMSO solution and shaken well to be extracted by DCM). LiCl was used to interact with DMSO to sep-arate it from the more polar hydrazone compound. The DCM was then used as the or-ganic layer in the solution to extract the hydrazone from the DMSO. The DMSO sample and equal volume (1.5 mL) of LiCl was added to the test tube and vortexed. DCM was then added to the test tube. The mixture was vortexed until combined. The organic layer was collected and placed into another labeled test tube. This procedure was performed 5 times for each sample.

Gas Chromatography Mass Spectroscopy of the Samples

Samples were diluted with 10 mL of DCM into labeled test tubes. Into each labeled autosampler vial, 2-3 drops of the diluted solution were added and filled to 1.5 mL with DCM. Samples were tested by gas-chromatography/time-of-flight mass spectrometry (GC-TOFMS).

DPPH radical scavenging assay of the Samples

The 2,2-diphenyl-1-picrylhydrazyl (DPPH) assay was conducted following earlier procedures where 200 mL of 50% aqueous ethanol was first incubated for 2 h at 37 °C. A 50 mL working solution of DPPH was made by dissolving DPPH in the incubated 50% aqueous ethanol to a concentration of 222 μM. This solution was stirred in the dark for 45 minutes to ensure that all the DPPH was dissolved. Stock solutions of the control compound (Trolox) and the experimental compounds (phenols and thiophenols) were prepared to a concentration of 10 mM in DMSO. The stock solutions were all diluted with 50% ethanol to a concentration of 200 μM. Then, 20 μL of each compound was first added to a clear flat bottom 96-well plate in quadruplicate. Control sets with no DPPH and proportional amounts of DMSO and 50% ethanol were used to determine the background absorbance. Sets with no compounds and proportional amounts of DPPH and DMSO in 50% ethanol were used as negative controls. Other than the background absorbance wells, 180 μL of the DPPH working solution was added to each well of the 96-well plate. A VersaMax UV-Vis plate reader with 519 nm and 37 °C parameters was used with the SoftMax Pro 5 software (Molecular Devices) to determine the scavenging of the DPPH radical by the investigated compounds. Readings were collected every 15 min for 60 min with the covered plate incubating at 37 °C in the dark between readings. The absorbance data was processed using the equation below, where Abs_c is the absorbance of the control and Abs_t is the absorbance of the test sample to obtain the percent radical scavenging values.

$$Percent\ Radical\ Scavenging = \frac{Abs_c - Abs_t}{Abs_c} * 100$$

The 60 min percent radical scavenging values were standardized against the 60 min Trolox percent radical scavenging value to obtain the final Trolox equivalent values for each compound.

$$Trolox\ equivalent = \frac{Percent\ Radical\ Scavenging}{Percent\ Radical\ Scavenging_{\ Trolox}}$$

ABTS radical scavenging assay of the Samples

Following an earlier procedure, the 2,2'-azino-bis(3-ethylbenzothiazoline-6-sulfonic acid (ABTS) assay was carried out to determine the radical scavenging activity of these hydrazones. The ABTS radical was generated by dissolving both the ABTS to the concentration of 7 mM and $K_2S_2O_8$ to the concentration of 2.45 mM in 4 mL of DI water for 16 to 24 h before running the assay and kept in the dark. 200 mL of 75 mM phosphate-buffered saline (PBS) with 50 mM NaCl at pH 7.4 was incubated for 2 h at 37 °C. A working solution of ABTS was made by

adding the concentrated ABTS to the incubated phosphate buffer until an absorbance of 0.70 to 0.85 was reached and kept in the dark. A stock solution of the control compound (Trolox) and the experimental compounds (phenols and thiophenols) were prepared in DMSO to a concentration of 50 mM. The stock solutions were diluted with ethanol to a 500 µM concentration. Then, 4 µL of each compound was added to a clear flat bottom 96-well plate in triplicate. Control sets with no ABTS and no compounds but with proportional amounts of phosphate buffer and DMSO in ethanol were used to determine background absorbance. Sets with no compounds and proportional amounts of ABTS and DMSO in ethanol were used as negative controls. Other than the background absorbance wells, 196 µL of the ABTS working solution was added to each well of the 96-well plate. A VersaMax UV-Vis plate reader with the parameters of 734 nm and 37 °C was used with the SoftMax Pro 5 software (Molecular Devices) to determine the scavenging of the ABTS radical by the investigated compounds. Readings were collected at 0, 6, and 12 min with the covered plate incubating at 37 °C in the dark between readings. The absorbance data was processed using the equation below, where Abs_c is the absorbance of the control and Abs_t is the absorbance of the test sample to obtain the percent radical scavenging values.

$$Percent\ Radical\ Scavenging = \frac{Abs_c - Abs_t}{Abs_c} * 100$$

The 12 min percent radical scavenging values were standardized against the 12 min Trolox percent radical scavenging value to obtain the final Trolox equivalent values for each compound.

$$Trolox\ equivalent = \frac{Percent\ Radical\ Scavenging}{Percent\ Radical\ Scavenging_{\ Trolox}}$$

Structural Identification and Spectral Data ofthe Compounds

The structural identification of the hydrazones was carried out using 1H, 13C and 19F (when applicable) NMR spectroscopy and high resolution mass spectrometry (HR-MS). All HR-MS data are within the 5 ppm limit for the difference as compared to the calculated values.

(*E*)-N,N-dimethyl-4-((2-(3-(trifluoromethyl)phenyl)hydrazono)methyl)aniline (1)

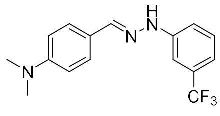

^{1}H NMR (400 MHz, DMSO-*d$_6$*) δ (ppm): 10.32 (s, 1H, NH), 7.85 (s, 1H, CH=), 7.51 (d, 2H, J = 8.0 Hz), 7.39 (t, 1H, J = 7.8 Hz), 7.34 (s, 1H), 7.27 (d, 1H, J = 7.9 Hz), 6.99 (d, 1H, J = 7.8 Hz), 6.71 (d, 2H, J = 4.1 Hz), 2.91 (s, 6H).

^{13}C NMR (100.58 MHz, DMSO-*d$_6$*) δ (ppm) : 151.2, 147.1, 140.2, 130.7 (q, C-CF_3, *J* = 31 Hz), 130.6, 127.8, 125.2 (q, CF_3, *J* = 273 Hz), 123.7, 115.9, 114.4, 112.6, 108.1 (q, C-C-CF_3, *J* = 4 Hz), 41.3

^{19}F NMR (376 MHz, DMSO-*d$_6$*) δ (ppm) : - 61.55

HRMS $C_{16}H_{16}F_3N_3$: Calc.: 307.12963 Found: 307.12853

(*E*)-1-benzylidene-2-phenylhydrazine (2)

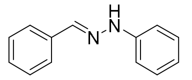

^{1}H NMR (300 MHz, $CDCl_3$), δ (ppm): 10.33 (s, 1H, NH), 7.86 (s, 1H, CH=), 7.63 (d, 2H, *J* = 4.1 Hz), 7.36 (t, 2H, *J* = 7.2 Hz), 7.27-7.19 (m, 3H), 7.08 (d, 2H, *J* = 4.0 Hz) 6.73 (t, 1H, *J* = 7.8 Hz)

^{13}C NMR (100.58 MHz, DMSO-*d$_6$*) δ (ppm) : 145.7, 136.8, 136.3, 129.6, 129.1, 128.3, 126.1, 119. 2, 112.4.

HRMS $C_{13}H_{12}N_2$: Calc.: 196.10005, Found: 196. 09898

(*E*)-1-benzylidene-2-(3-(trifluoromethyl)-phenylhydrazine (3)

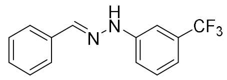

^{1}H NMR (400 MHz, DMSO-*d$_6$*) δ 10.68 (s, 1H, NH), 7.93 (s, 1H, CH=), 7.66 (d, 2H, *J* = 4.1 Hz), 7.41 – 7.25 (m, 6H), 7.02 (d, 1H, *J* = 4.5 Hz).

^{13}C NMR (100.58 MHz, DMSO-*d$_6$*) δ 146.4, 138.7, 135.8, 130.5 (q, C-CF_3, *J* = 30 Hz), 130.4, 129.0, 128.8, 126.3, 124.9 (q, CF_3, *J* = 272 Hz), 116.0, 115.1, 108.3.

^{19}F NMR (376 MHz, DMSO-*d$_6$*) δ (ppm) : - 61.6

HRMS $C_{14}H_{11}F_3N_2$: Calc.: 264.08743, Found: 264. 09200

(*E*)-1-benzylidene-2-(1,2,3,4,5-pentafluoro)-phenylhydrazine (4)

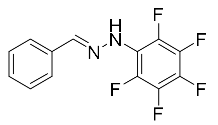

^{1}H NMR (400 MHz, DMSO-*d$_6$*) δ 10.28 (s, 1H, NH), 8.08 (s, 1H, CH=), 7.56 (d, 2H, *J* = 4.1 Hz), 7.36 (t, 2H, *J* = 8 Hz), 7.30 (t, 1H, *J* = 7.9 Hz).

^{13}C NMR (100.58 MHz, DMSO-*d$_6$*), δ 142.4, 139.3 (m), 136.7 (m), 135.2, 132.9 (m), 129.3, 129.1, 126.4, 121.7.

^{19}F NMR (376 MHz, DMSO-*d$_6$*) δ (ppm) : -156.0,- 164.5, - 170.3.

HRMS $C_{13}H_7F_5N_2$: Calc.: 286.05294; Found: 286. 04846

(*E*)-N,N-dimethyl-4-((2-phenyl)hydrazynylidene)methyl)aniline (5)

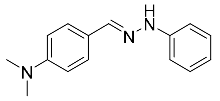

^{1}H NMR (400 MHz, DMSO-*d$_6$*) δ 9.04 (s, 1H, NH), 6.87 (s, 1H, CH=), 6.55 (d, 2H, *J* = 3.9 Hz), 6.27(t, 2H, *J* = 7.5 Hz), 6.13 (d, 2H, J = 4.0 Hz), 5.78 (m, 3H), 1.98 (s, 6H, CH_3).

^{13}C NMR (100.58 MHz, DMSO-*d$_6$*), δ 149.8, 145.4, 137.3, 126.7, 126.4, 123.2 117.4, 111.6, 111.2, 39.4.

HRMS $C_{15}H_{17}N_3$: Calc.: 239.14225 Found: 239.14263

(*E*)-4-((2-phenylhydrazono)methyl)phenol (6)

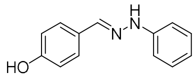

^{1}H NMR (300.12 MHz, $CDCl_3$), δ (ppm) 9.98 (s, 1H, NH), 9.61 (s, broad, 1H, OH), 7.76 (s, 1H, CH=), 7.45 (d, 1H, *J*= 4.0 Hz), 7.16 (t, 3H, *J*=4.1 Hz), 7.00 (d, 3H, *J* = 3.9 Hz), 6.78 (d, 1H, *J*= 4.2 Hz), 6.66 (t, 1H, *J*= 4.1 Hz).

^{13}C NMR (75.47 MHz, $CDCl_3$), (ppm) 158.1, 146.1, 137.5, 129.4, 127.6, 127.4, 118.5, 115.9, 112.1.

HRMS $C_{13}H_{12}N_2O$: Calc.: 212.09469; Found: 212. 10139

(*E*)-1-(3,4-dihydroxybenzylidene)-2-(3-(trifluoromethyl)-phenylhydrazine (7)

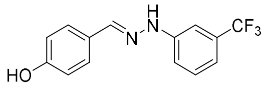

^{1}H NMR (400 MHz, DMSO-*d$_6$*) δ 10.39 (s, 1H, NH), 9.69 (s, 1H, OH), 7.85 (s, 1H, CH=), 7.51 (d, 2H, *J* = 4 Hz), 7.36 (t, 1H, *J* = 8.0 Hz), 7.31 (s, 1H), 7.24 (d, 1H, J = 3.9 Hz), 6.97 (d, 1H, *J* = 4.1 Hz), 6.82 (d, 2H, *J* = 8.3 Hz

^{13}C NMR (100.~~58~~ MHz, DMSO-*d$_6$*), δ 158.6, 146.7, 139.4, 130.4 (q, C-CF_3, *J* = 30 Hz), 130.4, 128.0, 124.9 (q, CF_3, *J* = 271 Hz), 126.8, 116.1, 115.8, 114.4, 107.9.

^{19}F NMR (376 MHz, DMSO-*d$_6$*) δ (ppm) : - 61.6.

HRMS $C_{14}H_{11}F_3N_2O$: Calc.: 280.08235; Found: 280. 08752

(*E*)-1-(3,4-dihydroxybenzylidene)-2-phenylhydrazine (8)

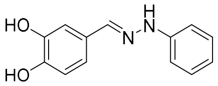

^{1}H NMR (400 MHz, DMSO-*d6*) δ 9.07 (s, 1H, NH), 8.93 (s, 2H, OH), 7.73 (s, 1H, CH=), 7.21 – 7.19 (s, 1H), 7.14 (m, 4H), 7.12 (d, 1H, J = 8.2 Hz), 6.97 (t, 1H, *J* = 8.0 Hz), 6.76 (t, 1H, J=7.9 Hz).

^{13}C NMR (100.58 MHz, DMSO-*d6*) δ 146.6, 146.2, 146.0, 137.9, 129.5, 127.8, 118.9, 118.5, 116.0, 112.3, 112.1

HRMS $C_{13}H_{10}N_2O_2$: Calc.: 226.07423; Found: 226.07351

(*E*)-1-(3,4-dimethoxybenzylidene)-2-phenylhydrazine (9)

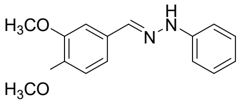

^{1}H NMR (400 MHz, DMSO-*d*$_6$), δ (ppm) 10.15 (s, 1H, NH), 7.80 (s, 1H, CH=), 7.30 (s, 1H),

7.19 (t, 1H, *J* = 4.1 Hz), 7.06 (d, 3H, *J* = 4 Hz), 6.92 (d, 2H, *J* = 4 Hz), 6.70 (t, 1H, *J* = 8.2 Hz), 3.79 (s, 3H), 3.74 (s, 3H).

^{13}C NMR (100.58 MHz, DMSO-*d*$_6$), δ (ppm) 149.6, 149.5, 146.0, 137.2, 129.5, 129.2, 119.9, 118.8, 112.3, 112.0, 108.1, 56.3, 55.9

HRMS $C_{15}H_{16}N_2O_2$: Calc.: 256.12118 Found: 256.12112

HRMS Spectra

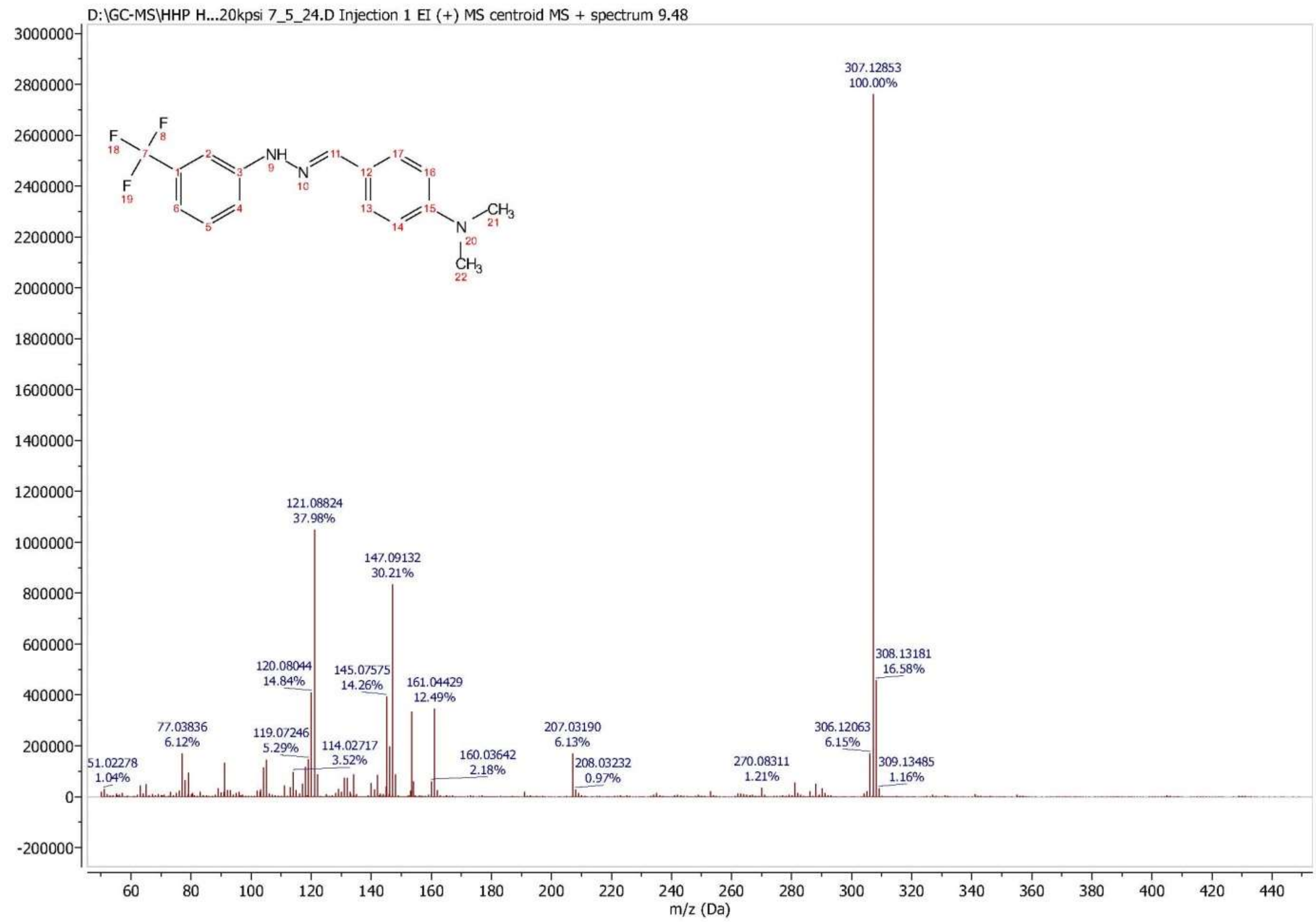

Figure S1. HRMS Spectrum of (*E*)-N,N-dimethyl-4-((2-(3-(trifluoromethyl)phenyl)hydrazono)-methyl)aniline (**1**)

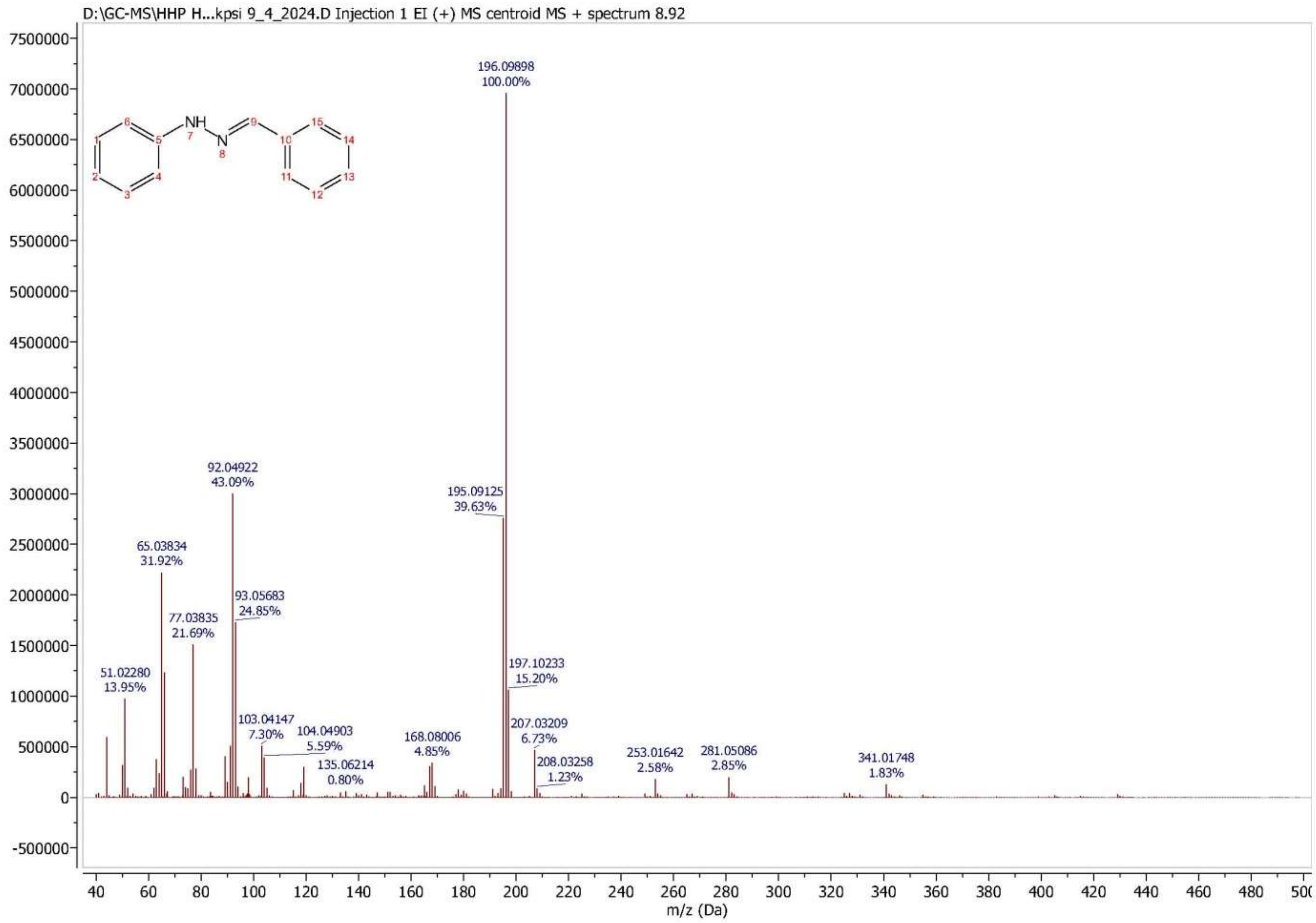

Figure S2. HRMS Spectrum of (*E*)-1-benzylidene-2-phenylhydrazine (**2**)

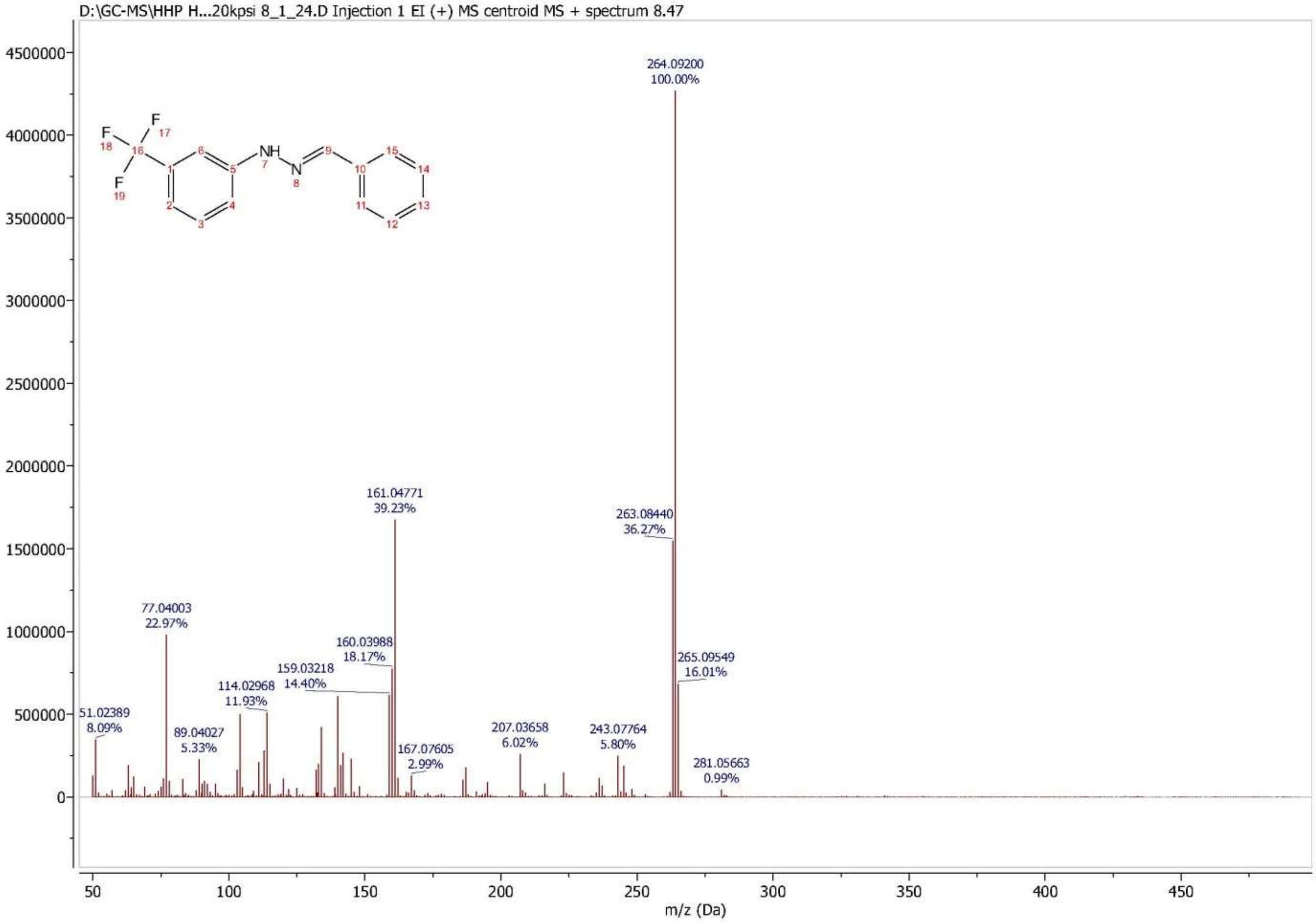

Figure S3. HRMS Spectrum of (*E*)-1-benzylidene-2-(3-(trifluoromethyl)-phenylhydrazine (**3**)

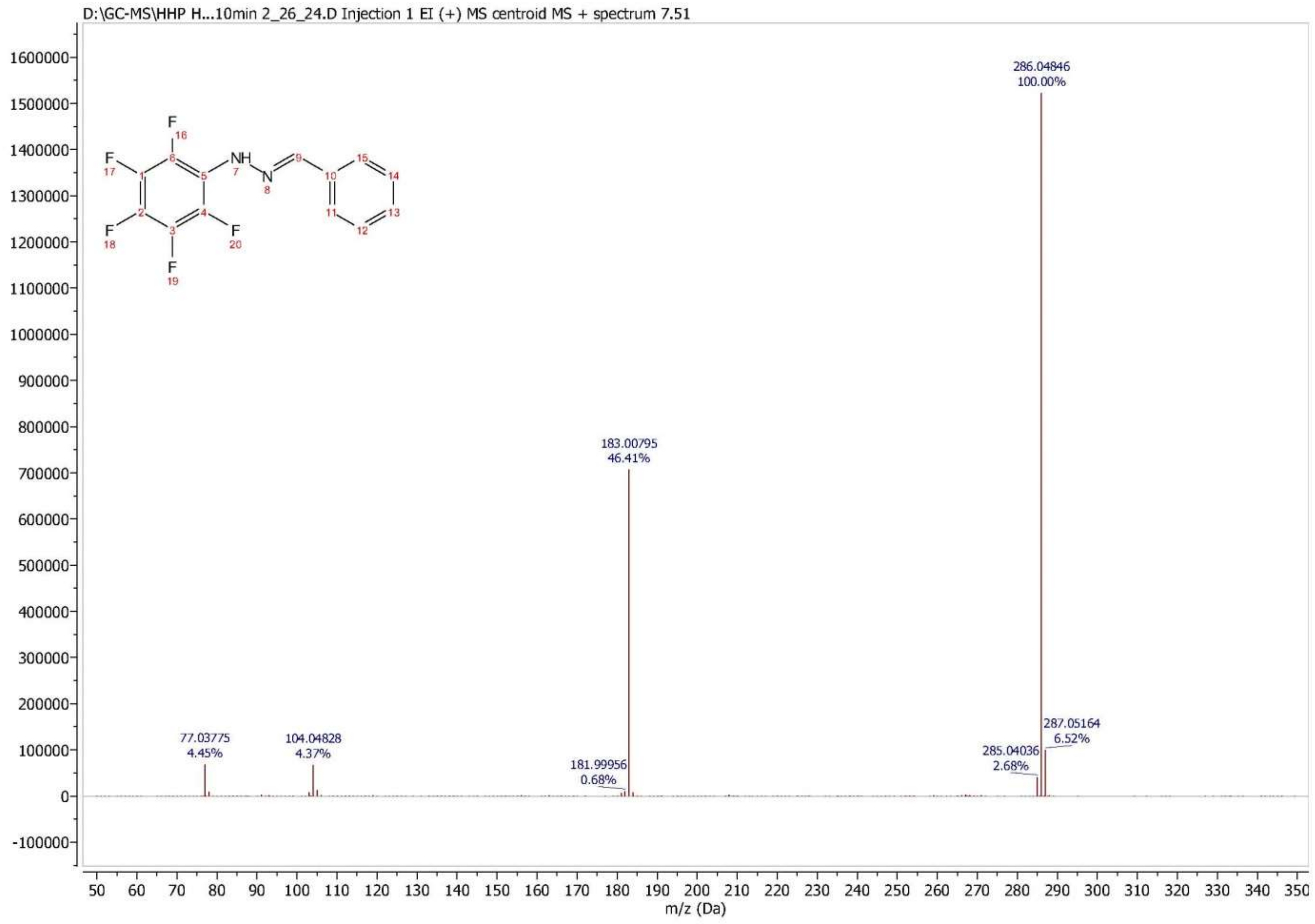

Figure S4. HRMS Spectrum of (*E*)-1-benzylidene-2-(1,2,3,4,5-pentafluoro)-phenylhydrazine (**4**)

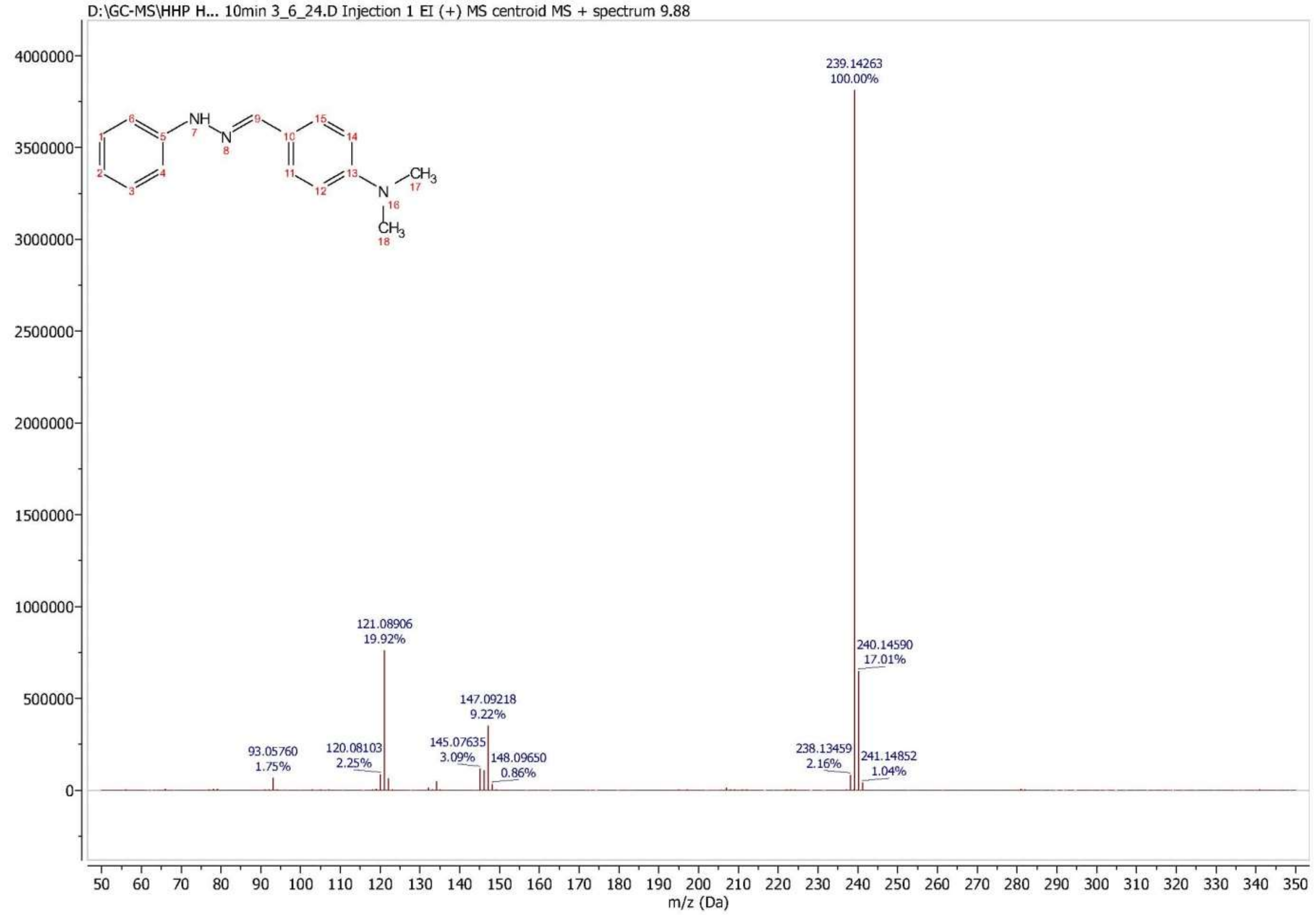

Figure S5. HRMS Spectrum of (*E*)-N,N-dimethyl-4-((2-phenyl)hydrazynylidene)methyl)aniline (**5**)

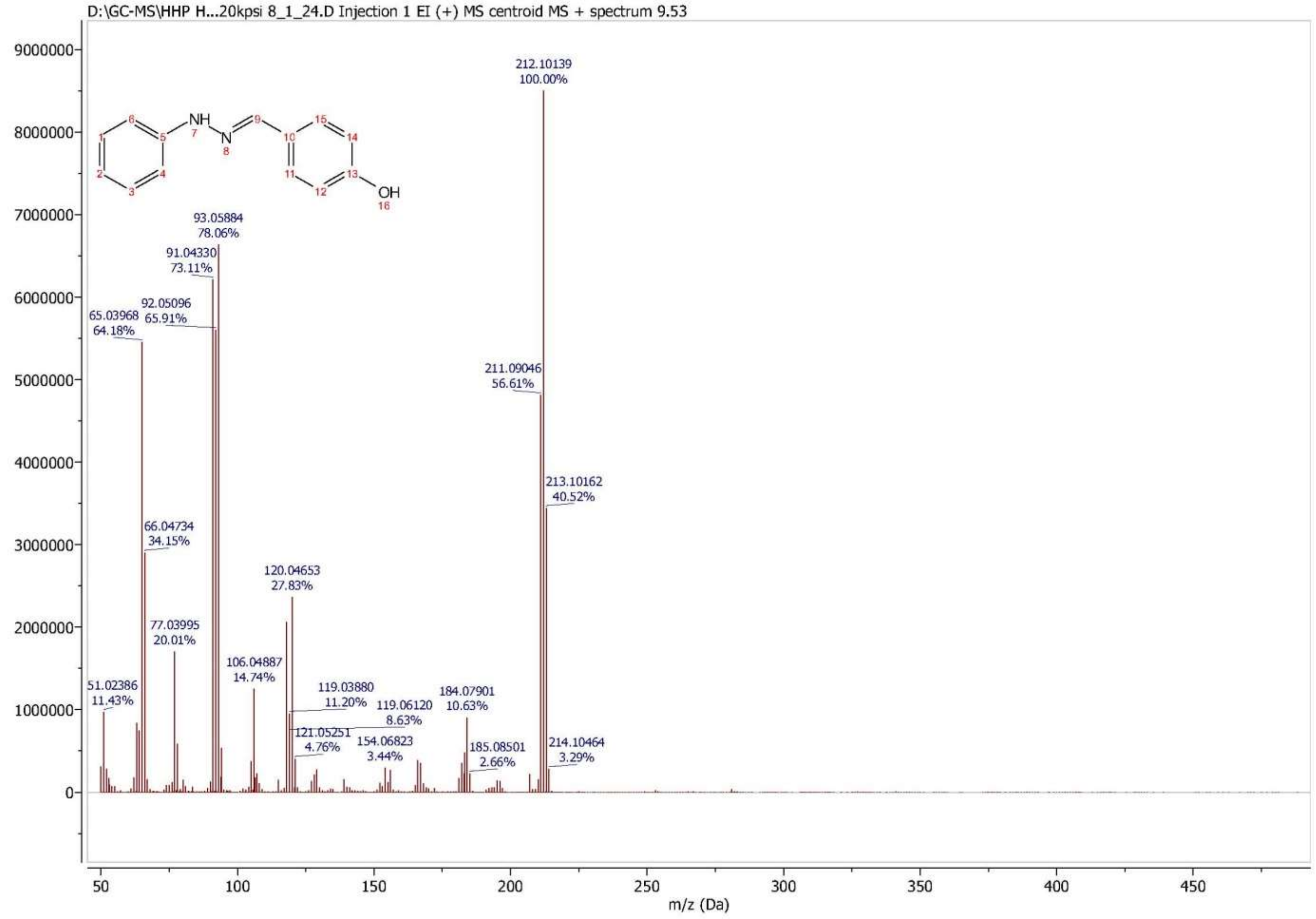

Figure S6. HRMS Spectrum of (*E*)-4-((2-phenylhydrazono)methyl)phenol (**6**)

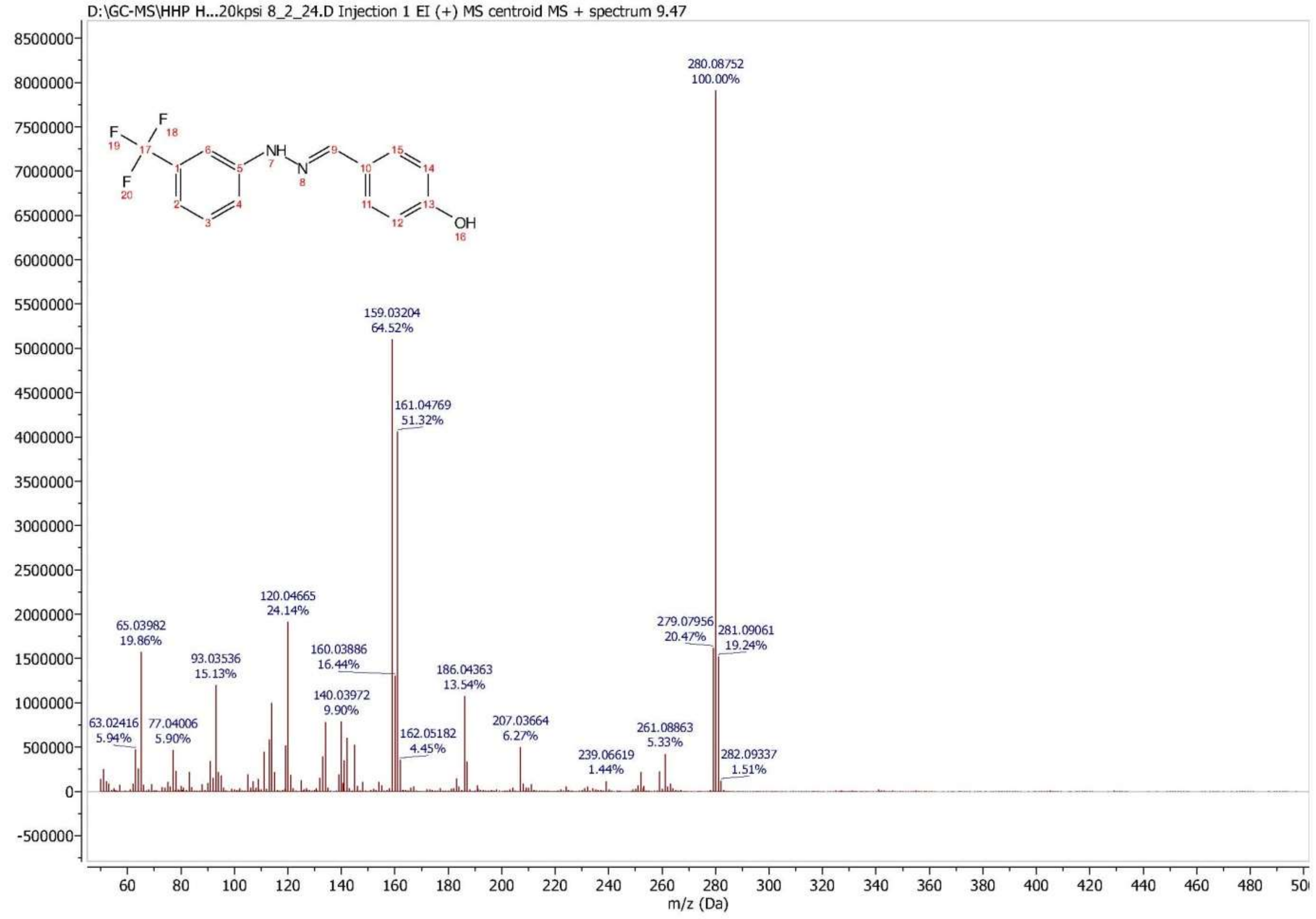

Figure S7. HRMS Spectrum of (*E*)-1-(3,4-dihydroxybenzylidene)-2-(3-(trifluoromethyl)-phenylhydrazine (**7**)

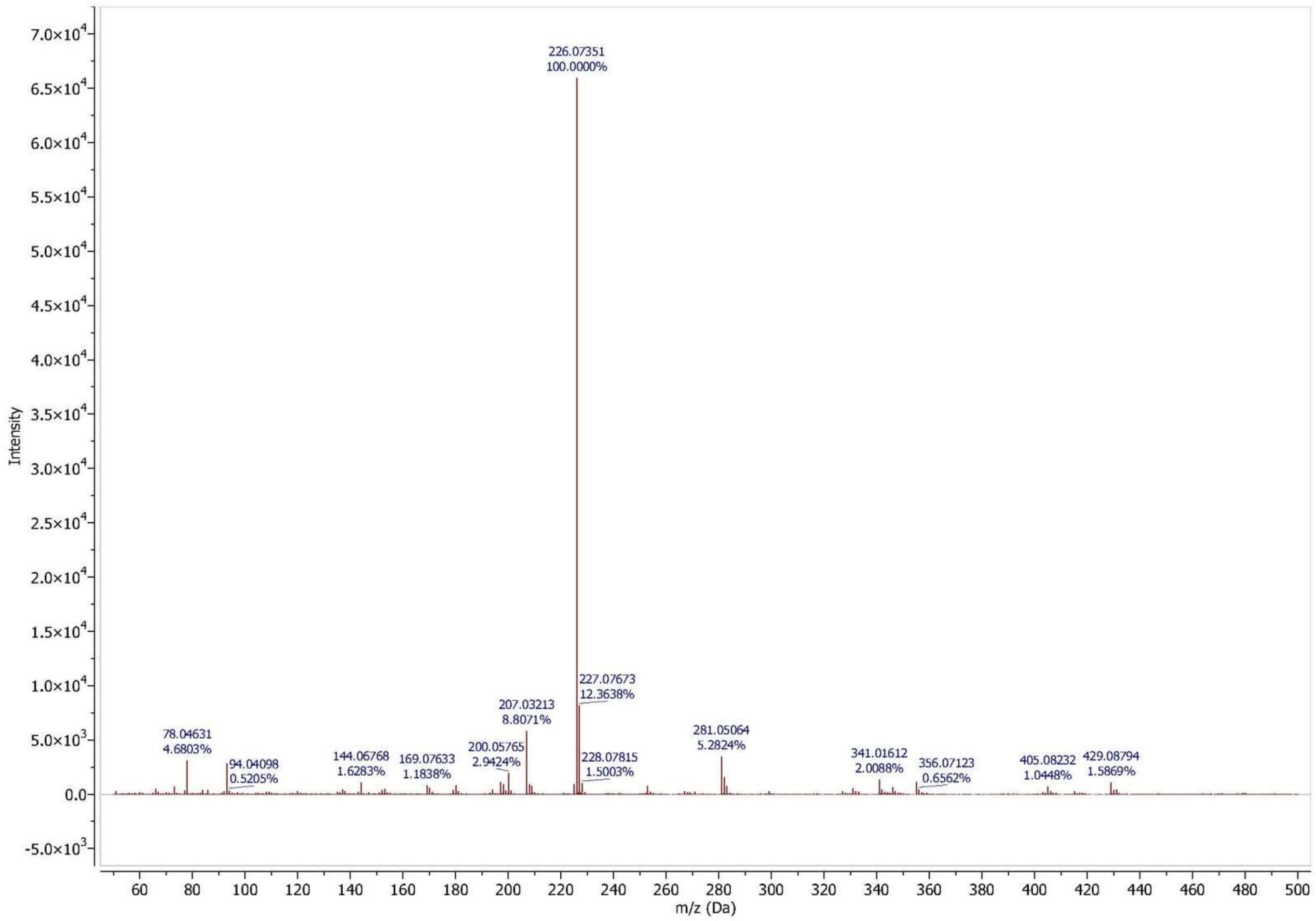

Figure S8. HRMS Spectrum of (*E*)-1-(3,4-dihydroxybenzylidene)-2-phenylhydrazine (**8**)

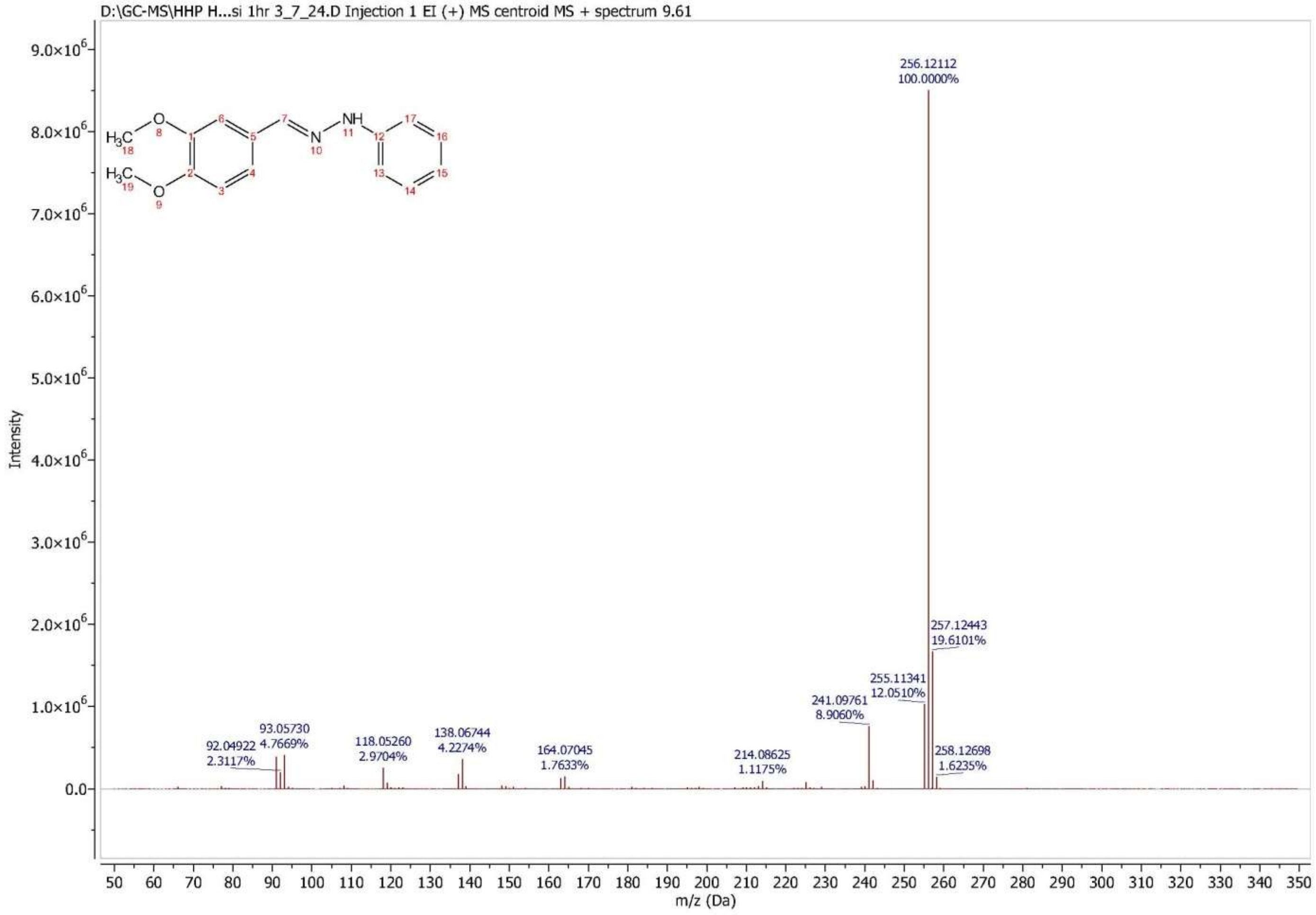

Figure S9. HRMS Spectrum of (*E*)-1-(3,4-dimethoxybenzylidene)-2-phenylhydrazine (**9**)

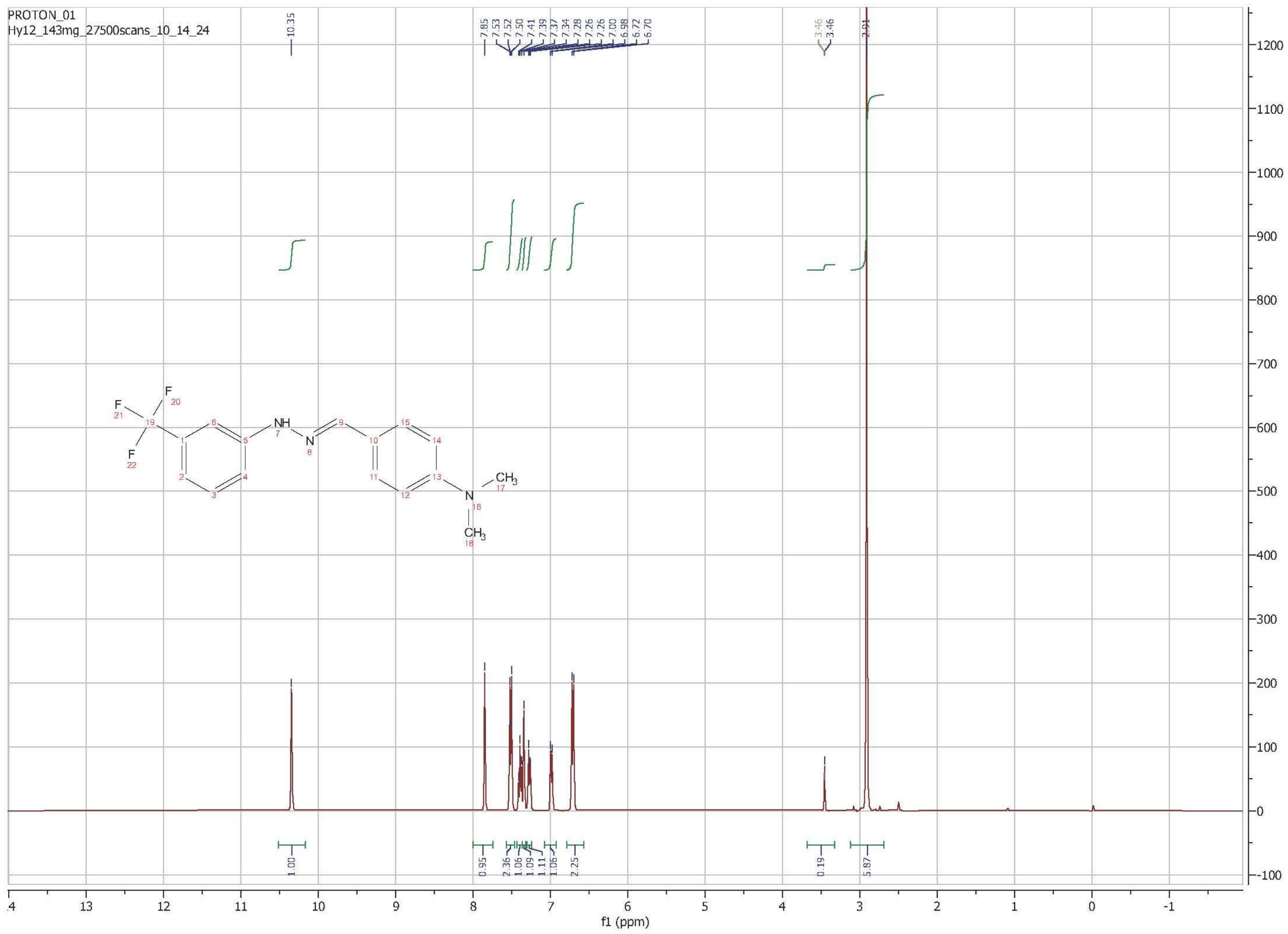

Fig. S10. ^{1}H NMR spectrum of (*E*)-N,N-dimethyl-4-((2-(3-(trifluoromethyl)phenyl)hydrazono)methyl)aniline (**1**).

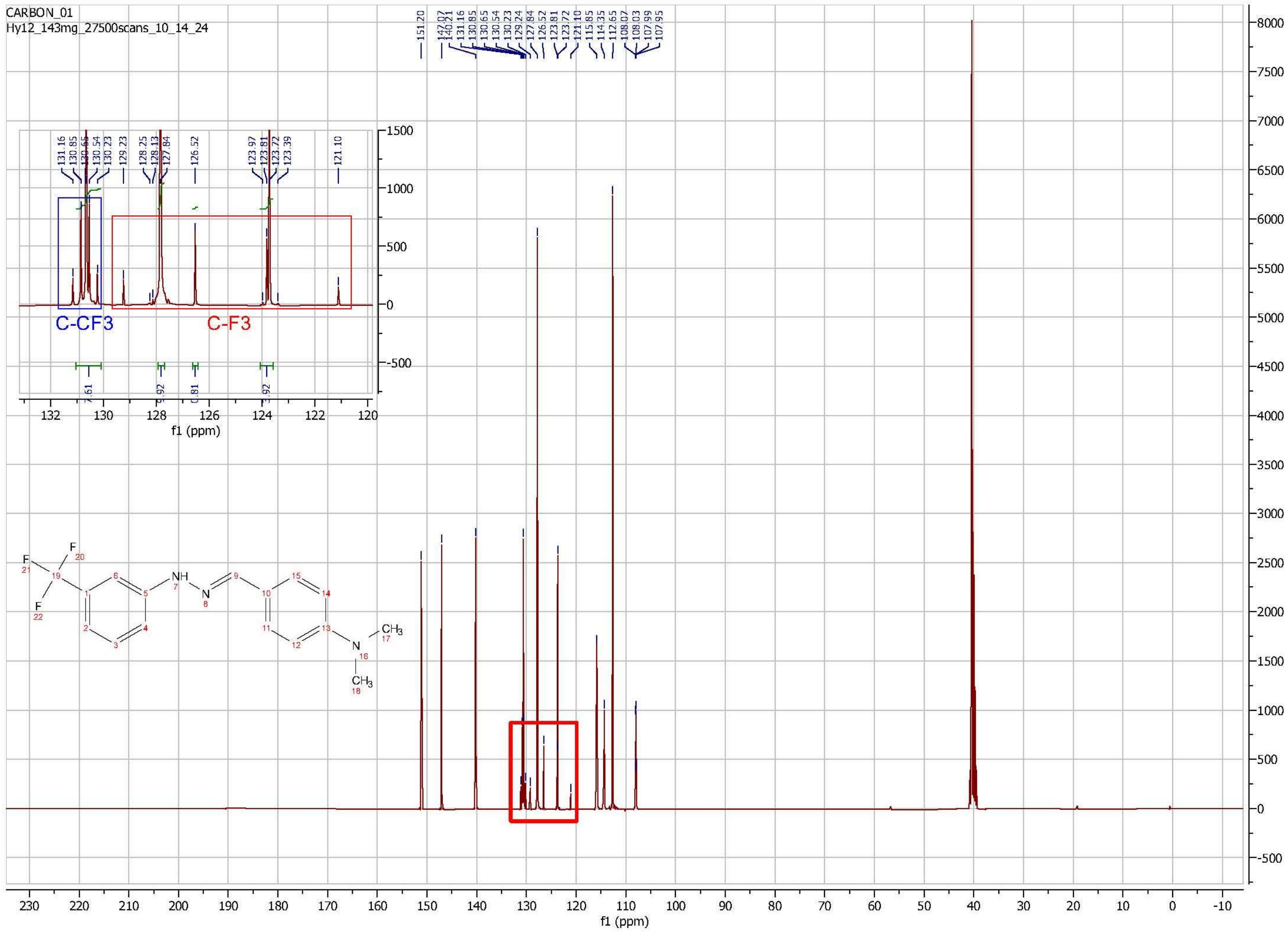

Fig. S11. ^{13}C NMR spectrum of (*E*)-N,N-dimethyl-4-((2-(3-(trifluoromethyl)phenyl)hydrazono)methyl)aniline (**1**).

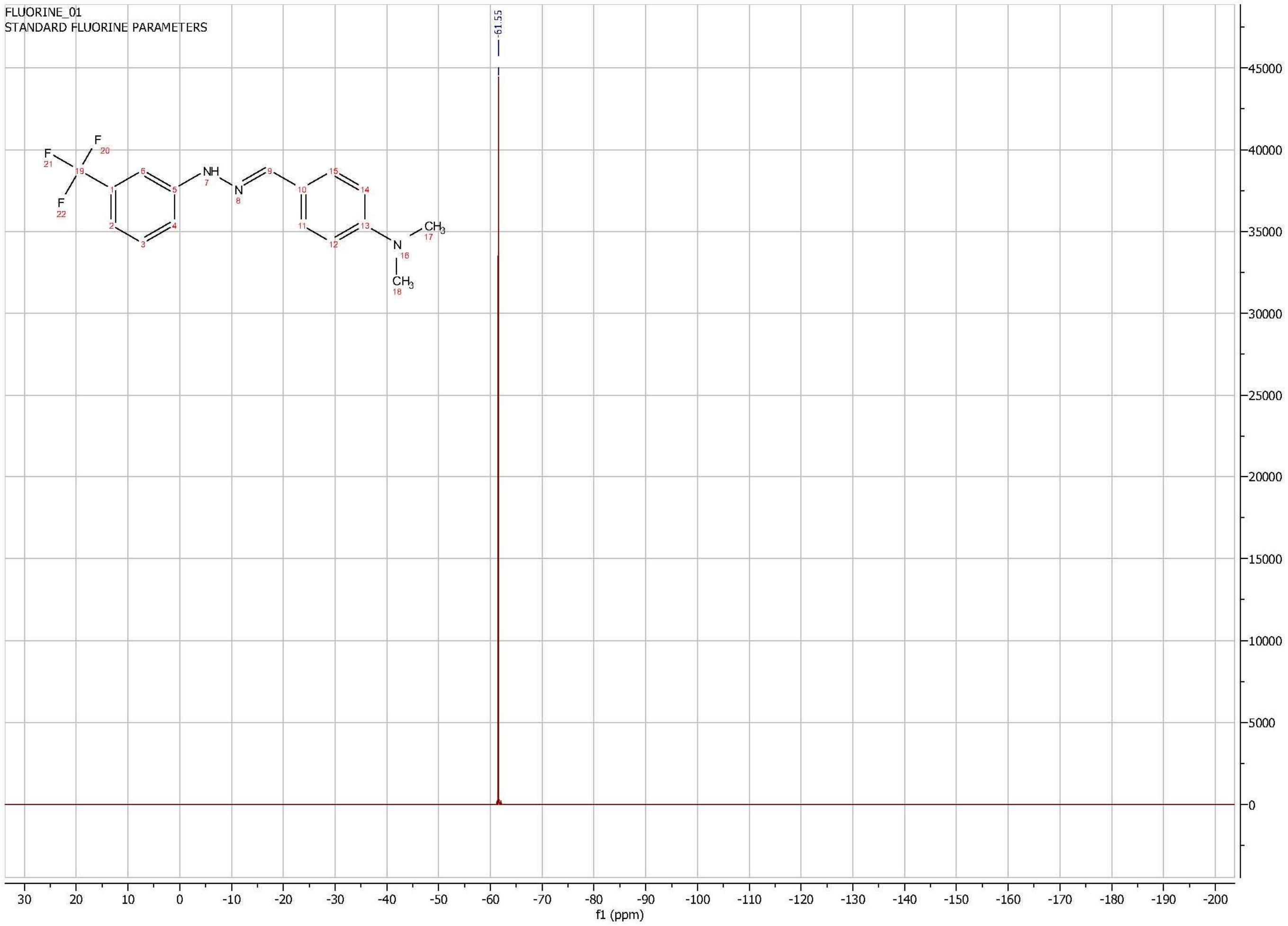

Fig. S12. ^{19}F NMR spectrum of (*E*)-N,N-dimethyl-4-((2-(3-(trifluoromethyl)phenyl)hydrazono)methyl)aniline (**1**).

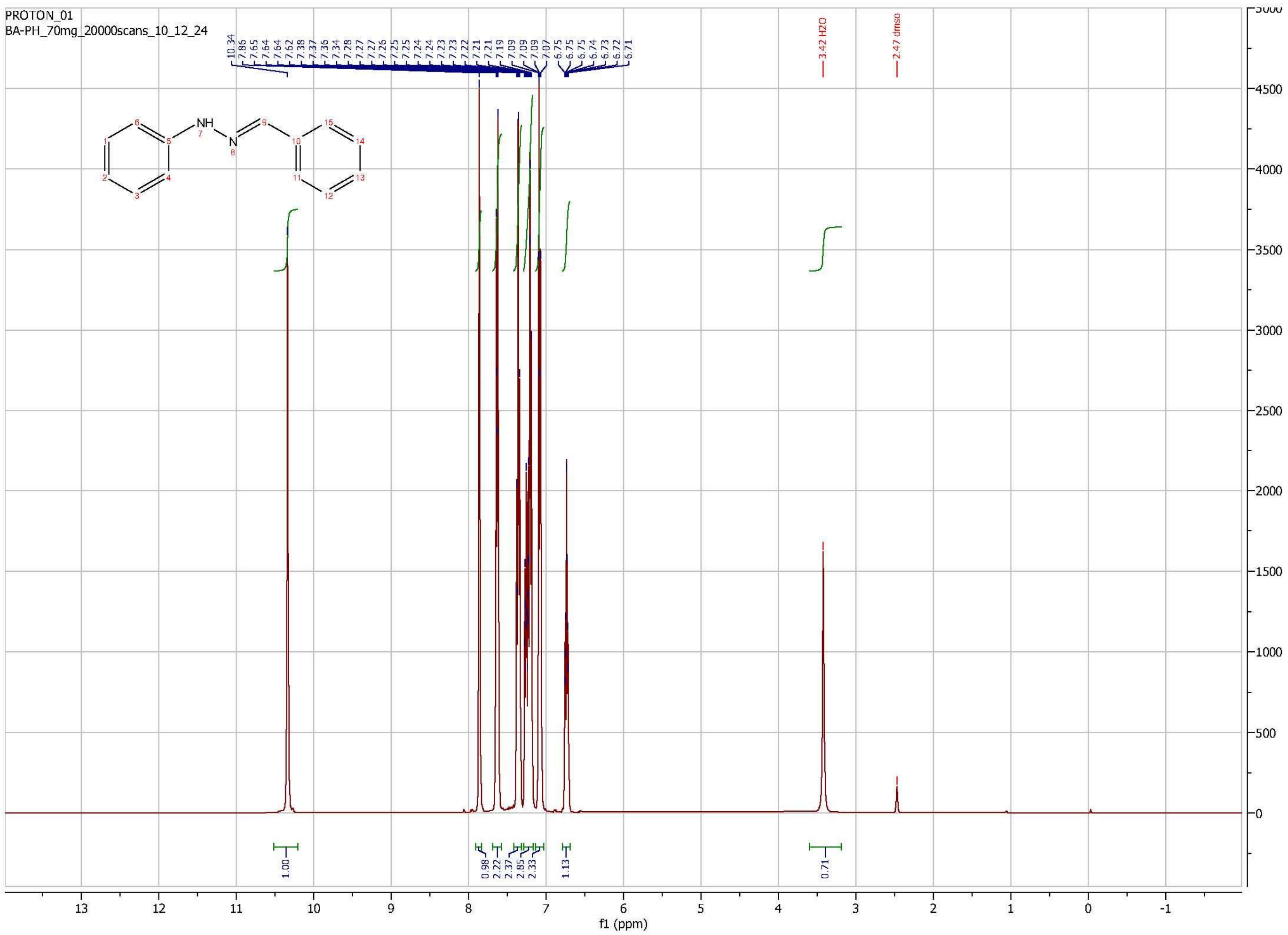

Fig. S13. ^{1}H NMR spectrum of (*E*)-1-benzylidene-2-phenylhydrazine (**2**).

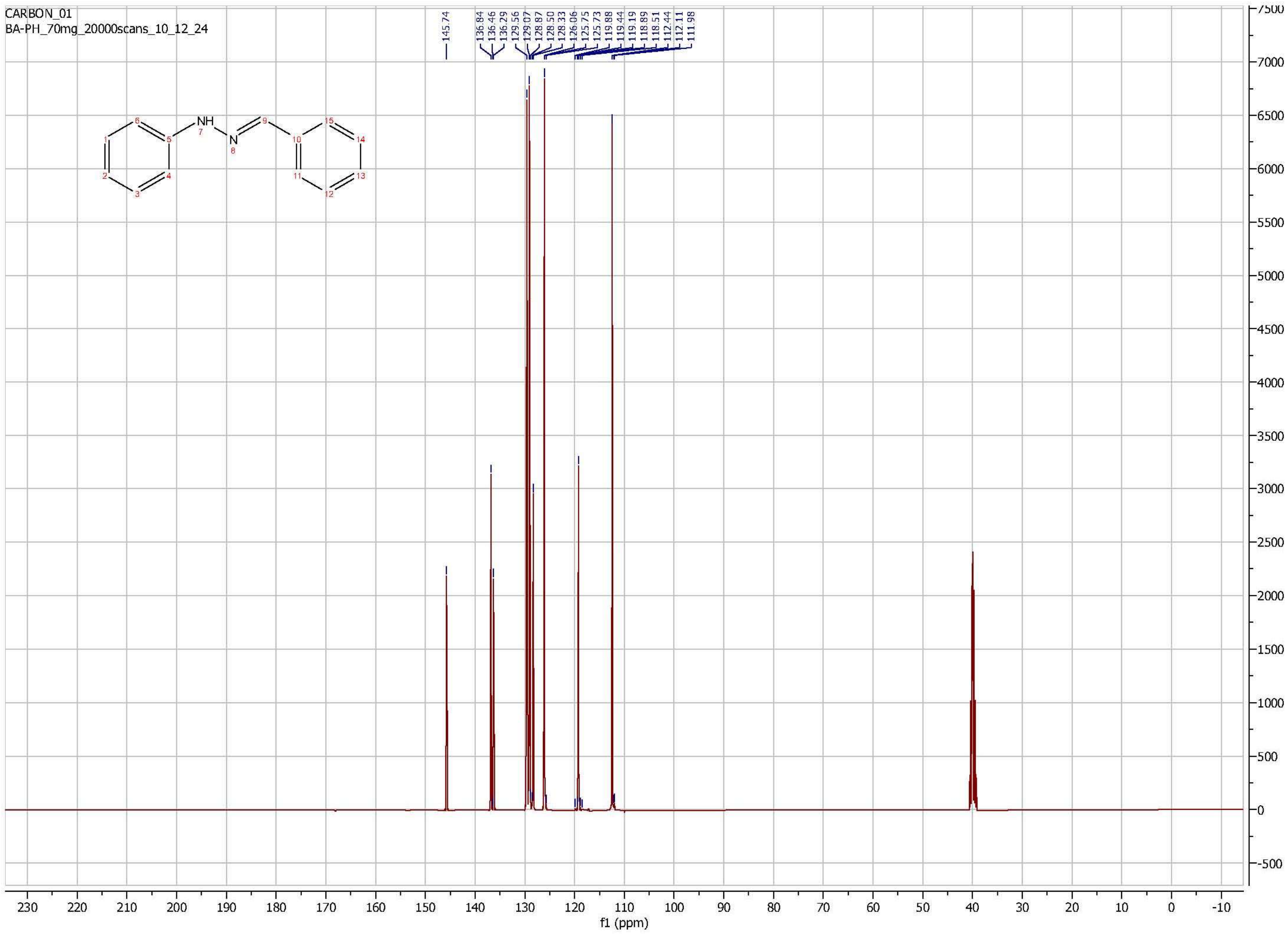

Fig. S14. ^{13}C NMR spectrum of (*E*)-1-benzylidene-2-phenylhydrazine (**2**).

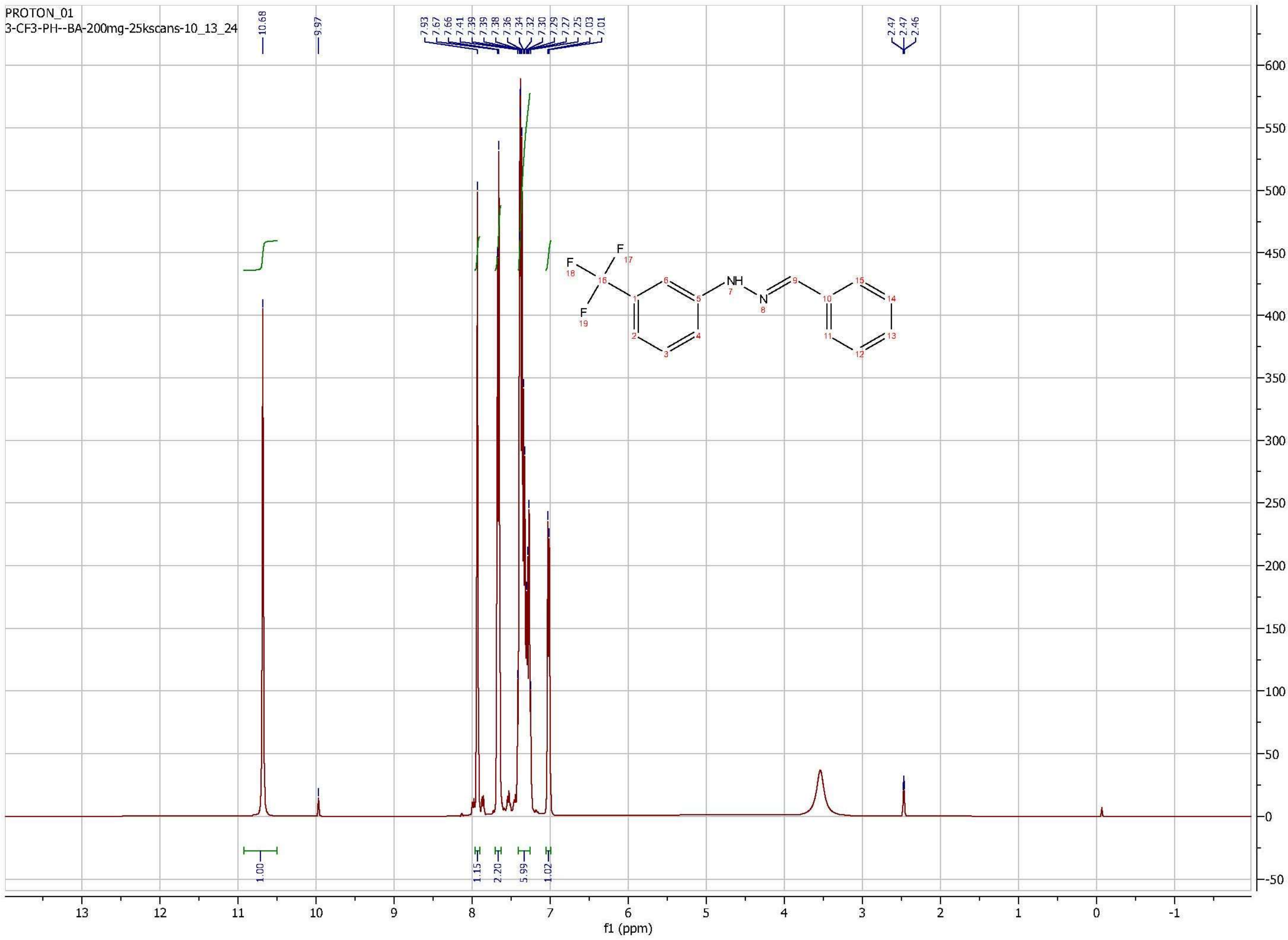

Fig. S15. ^{1}H NMR spectrum of (*E*)-1-benzylidene-2-(3-(trifluoromethyl)-phenylhydrazine (**3**)

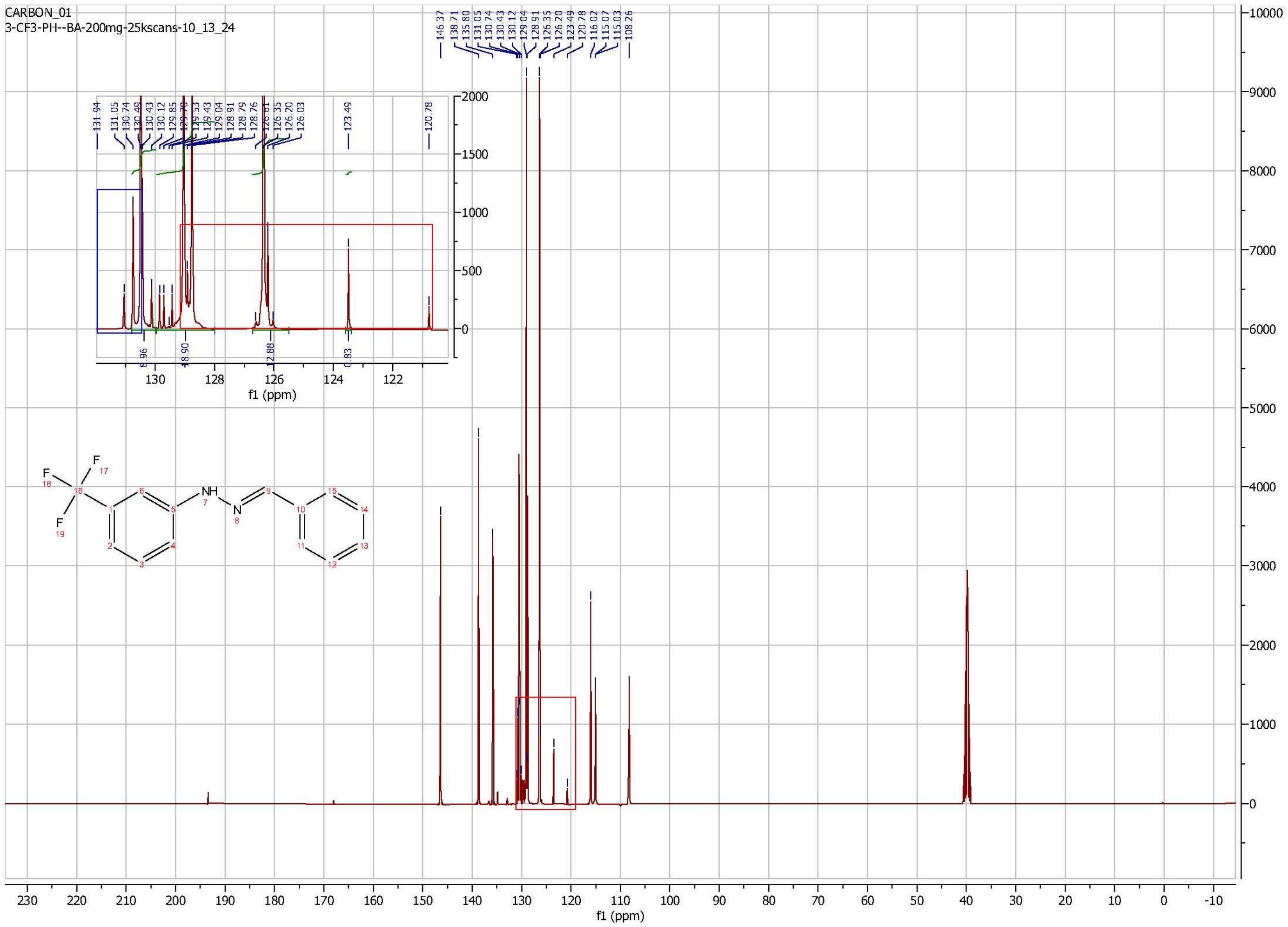

Fig. S16. ^{13}C NMR spectrum of (*E*)-1-benzylidene-2-(3-(trifluoromethyl)-phenylhydrazine (**3**)

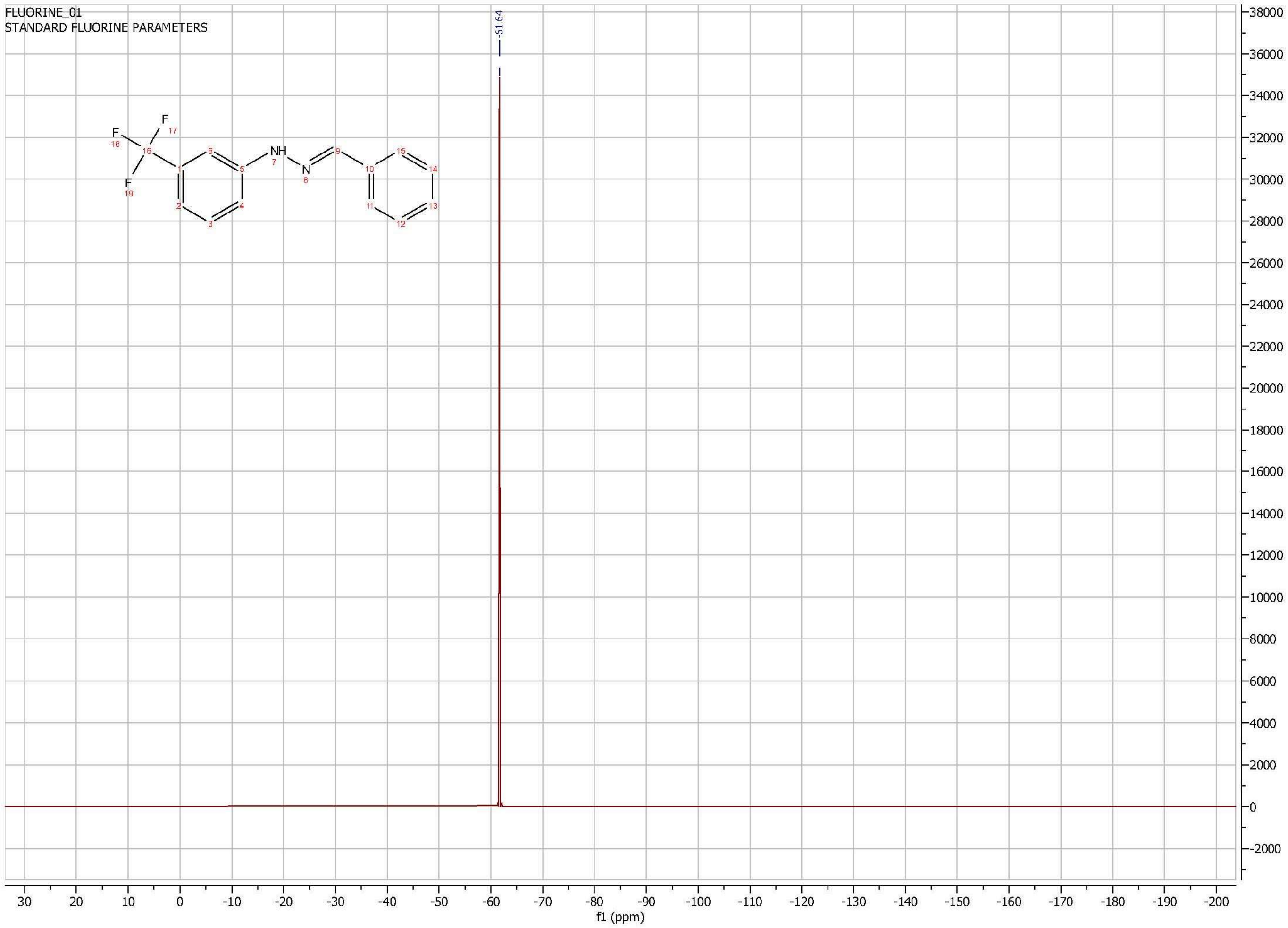

Fig. S17. ^{19}F NMR spectrum of (*E*)-1-benzylidene-2-(3-(trifluoromethyl)-phenylhydrazine (**3**)

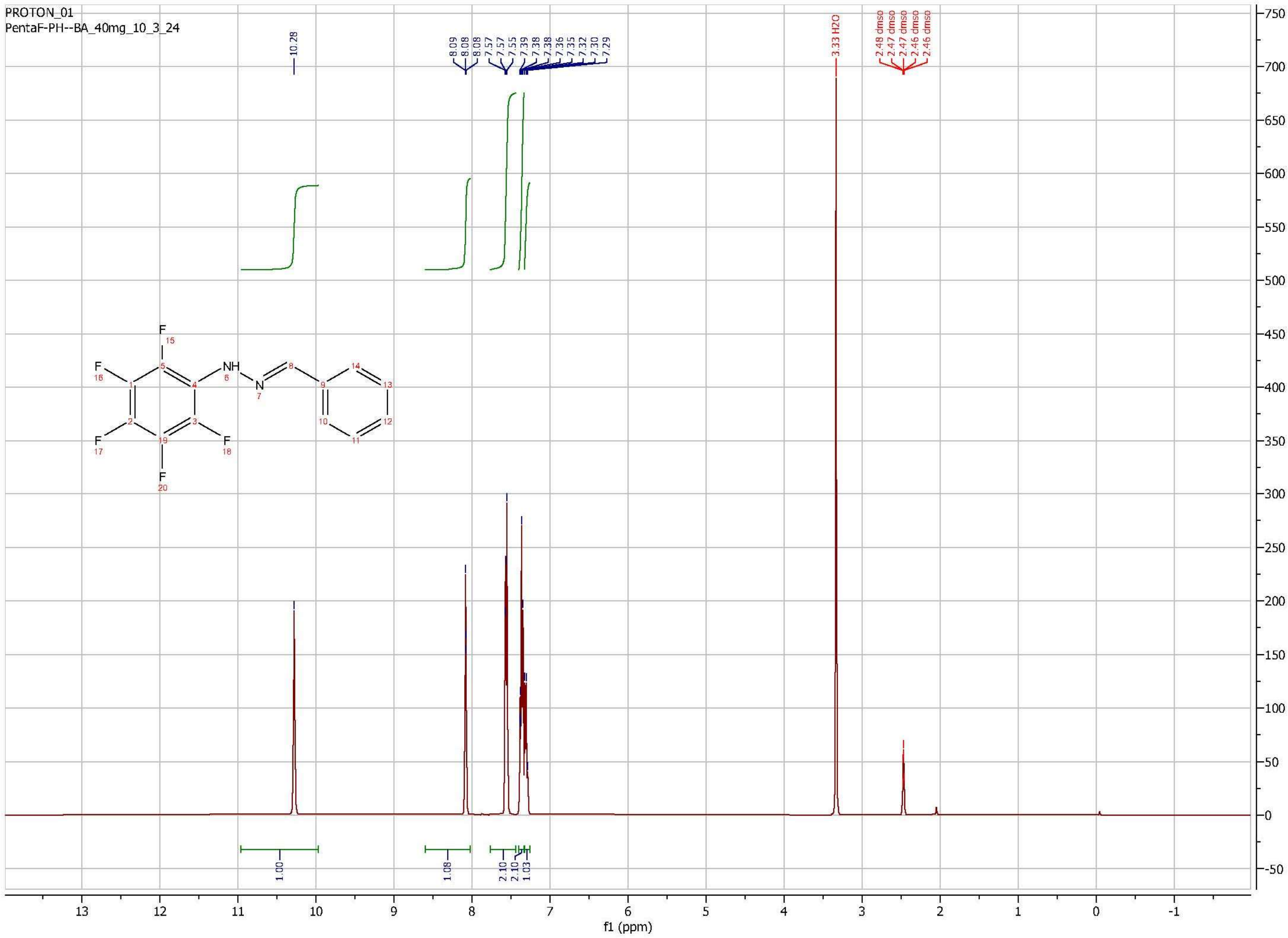

Fig. S18. ^{1}H NMR spectrum of (*E*)-1-benzylidene-2-(1,2,3,4,5-pentafluoro)-phenylhydrazine (**4**)

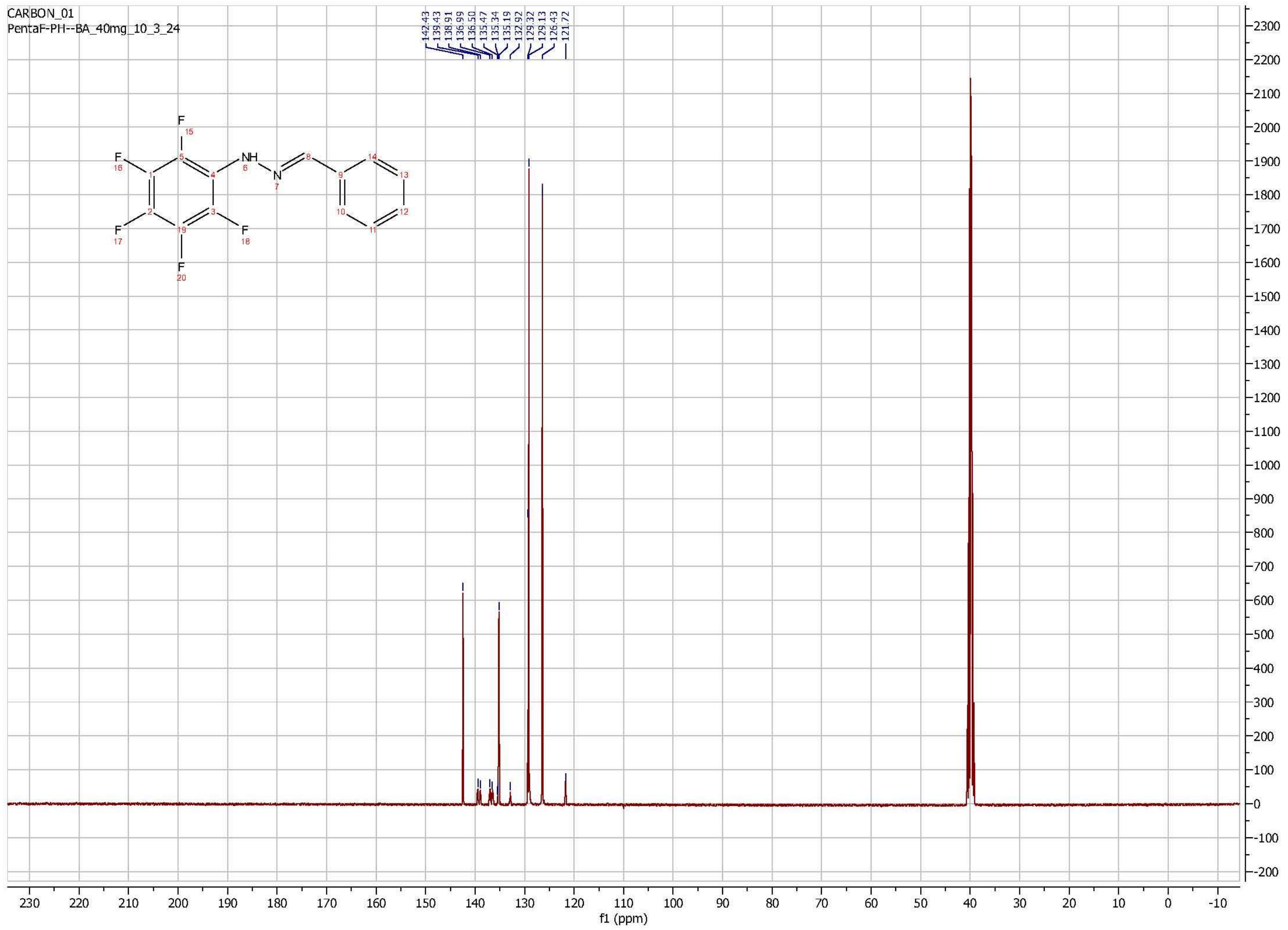

Fig. S19. ^{13}C NMR spectrum of (*E*)-1-benzylidene-2-(1,2,3,4,5-pentafluoro)-phenylhydrazine (**4**).

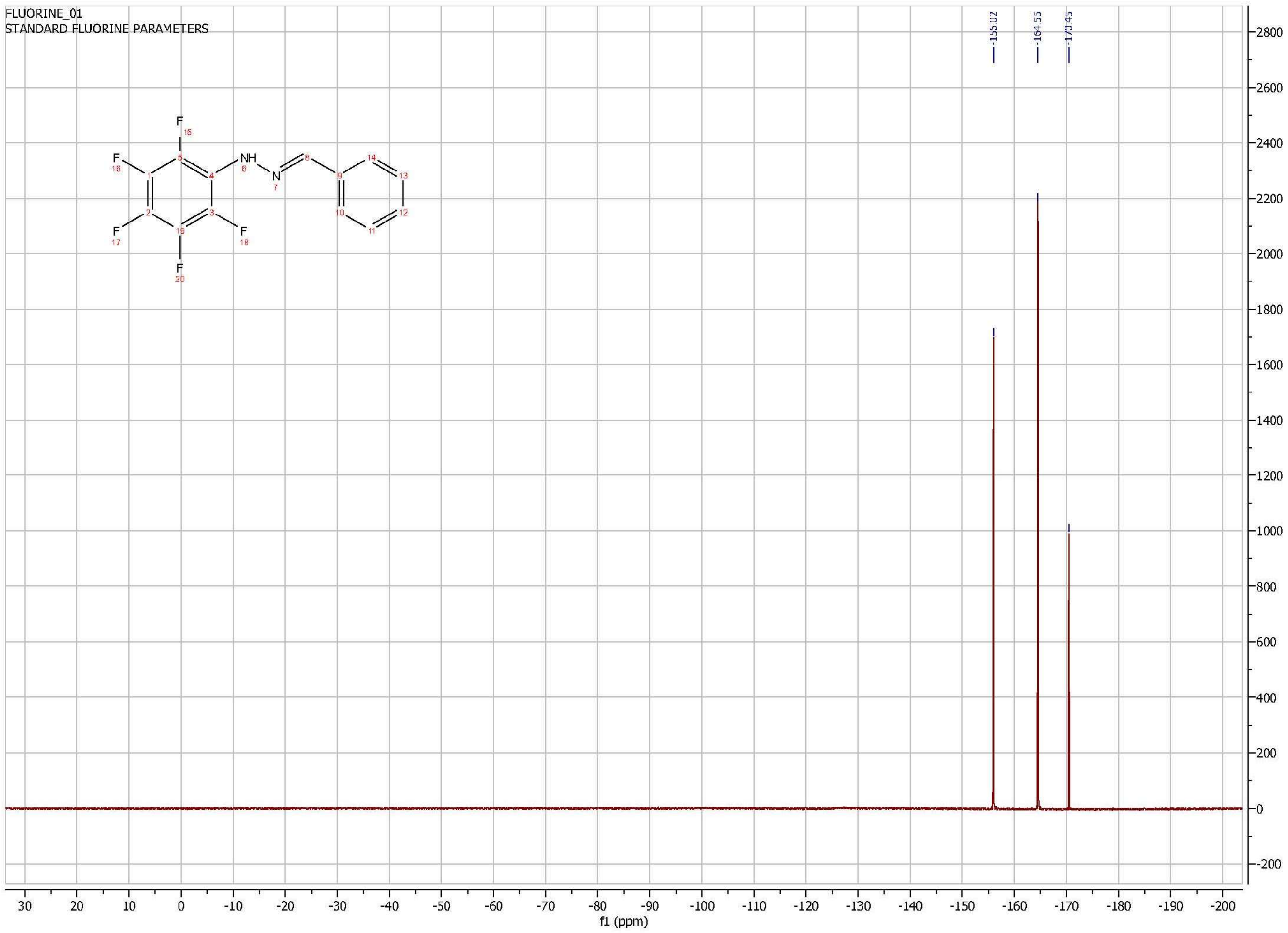

Fig. S20. ^{19}F NMR spectrum of (*E*)-1-benzylidene-2-(1,2,3,4,5-pentafluoro)-phenylhydrazine (**4**).

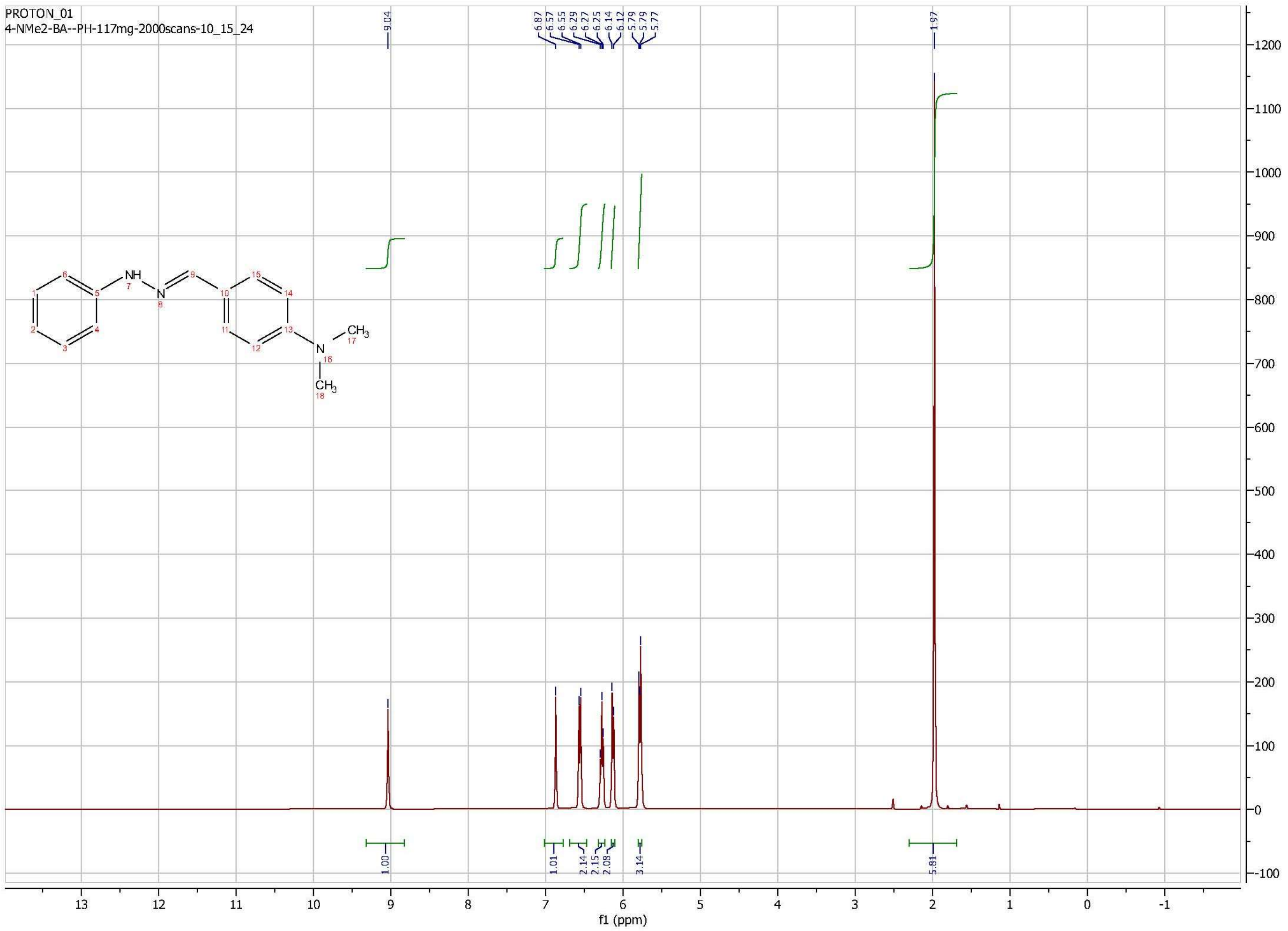

Fig. S21. ^{1}H NMR spectrum of (*E*)-N,N-dimethyl-4-((2-phenyl)hydrazynylidene)methyl)aniline (**5**)

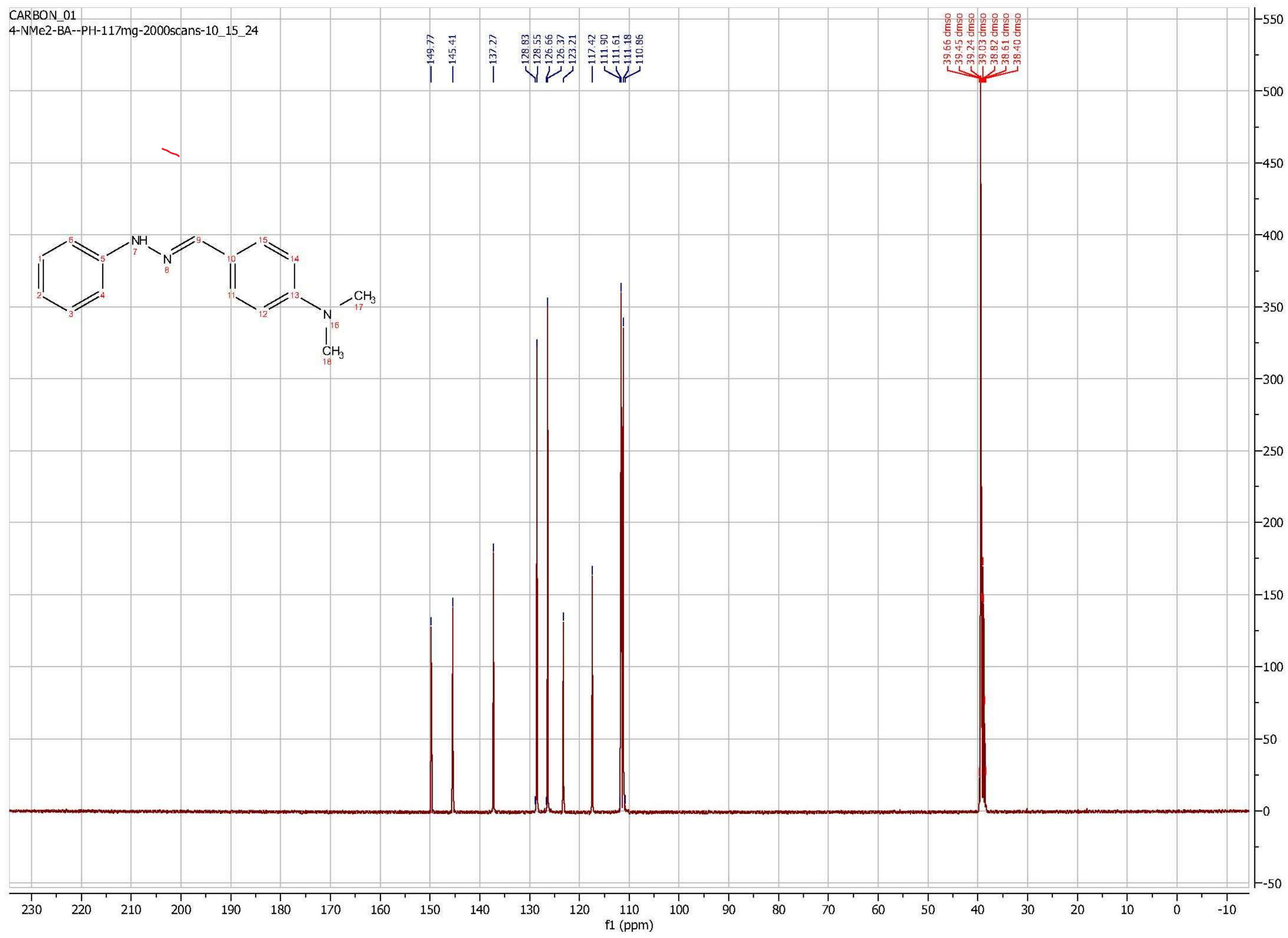

Fig. S22. ^{13}C NMR spectrum of (*E*)-N,N-dimethyl-4-((2-phenyl)hydrazynylidene)methyl)aniline (**5**).

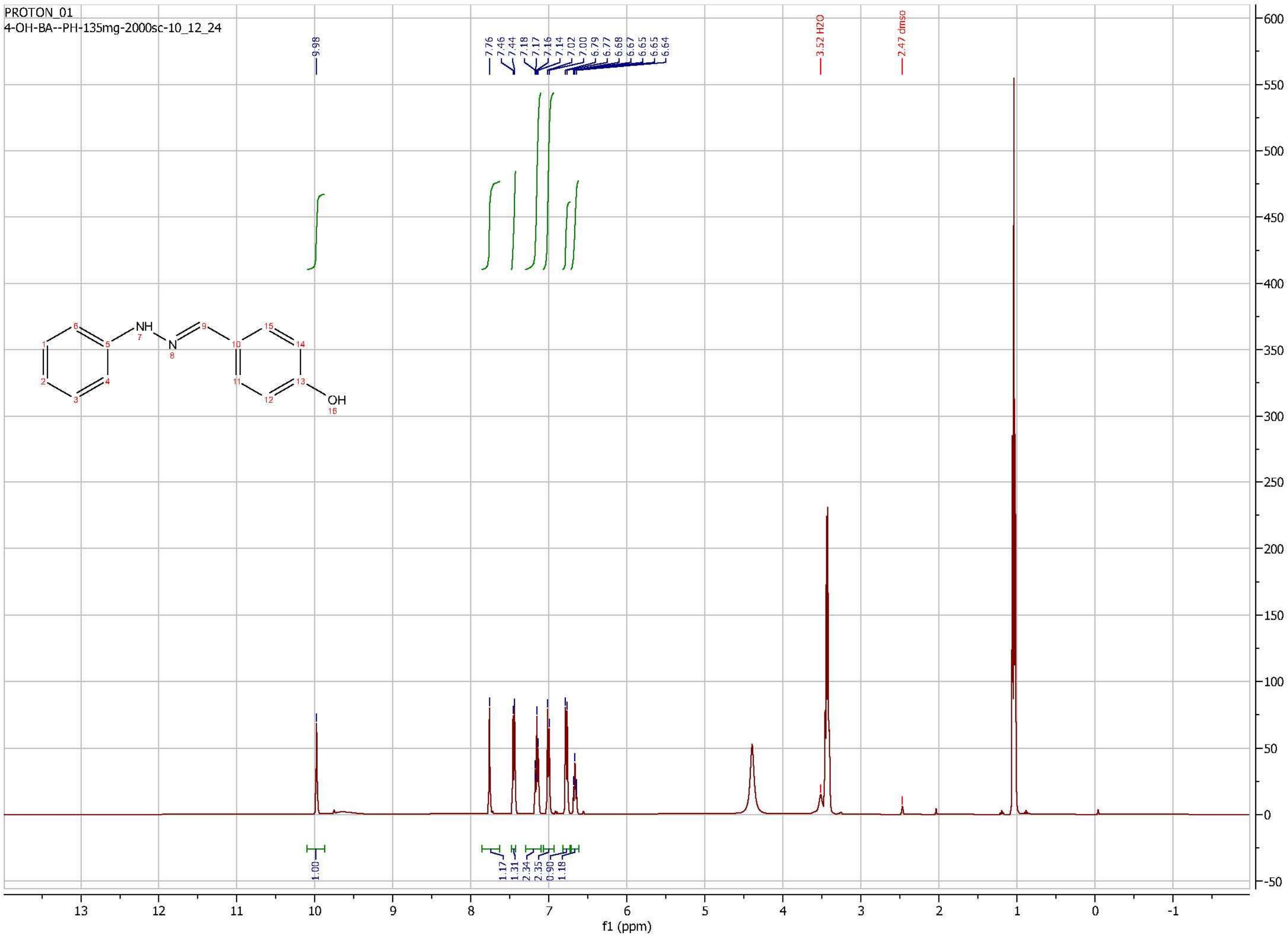

Fig. S23. ^{1}H NMR spectrum of (*E*)-4-((2-phenylhydrazono)methyl)phenol (**6**).

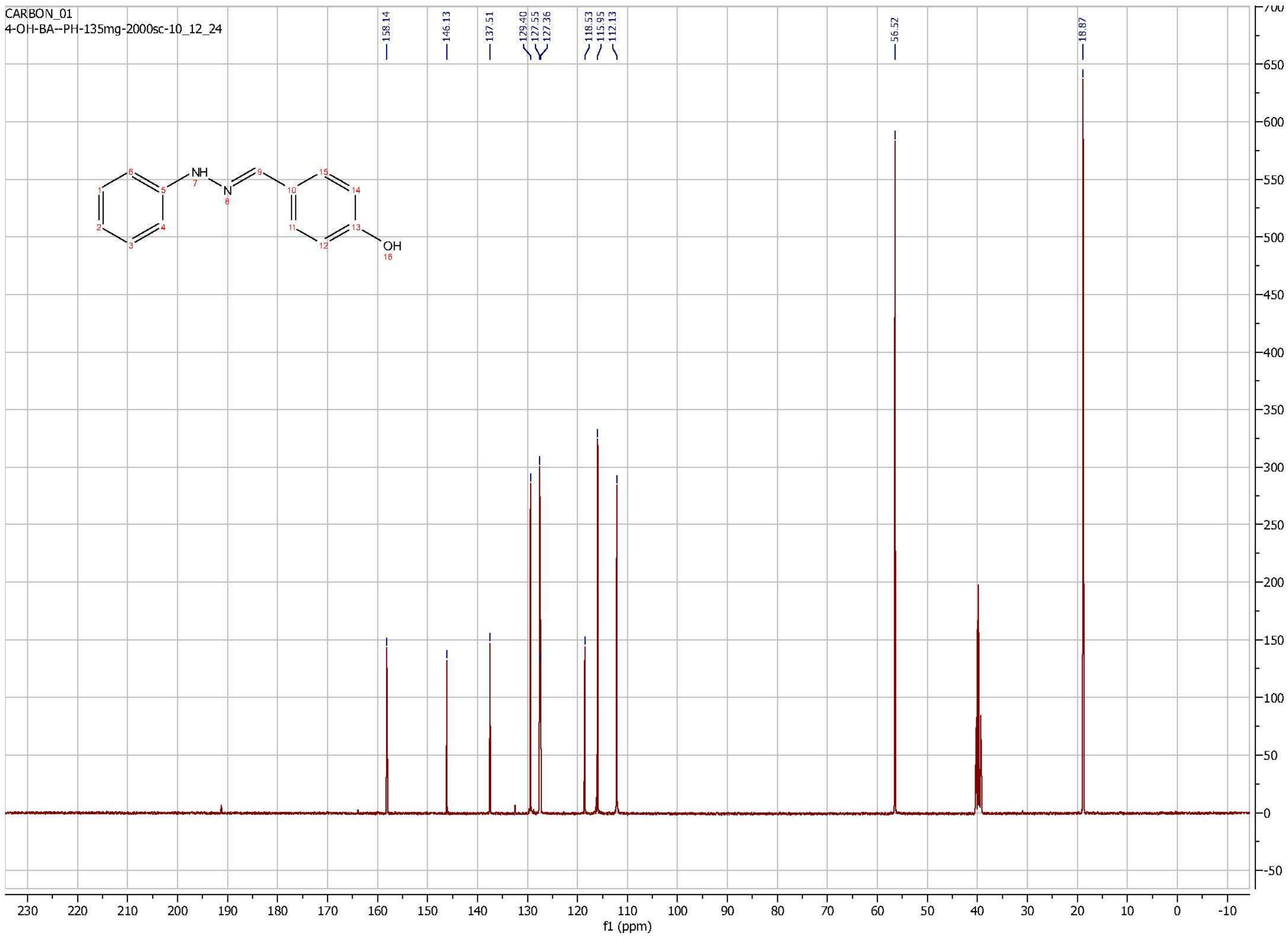

Fig. S24. ^{13}C NMR spectrum of (*E*)-4-((2-phenylhydrazono)methyl)phenol (**6**).

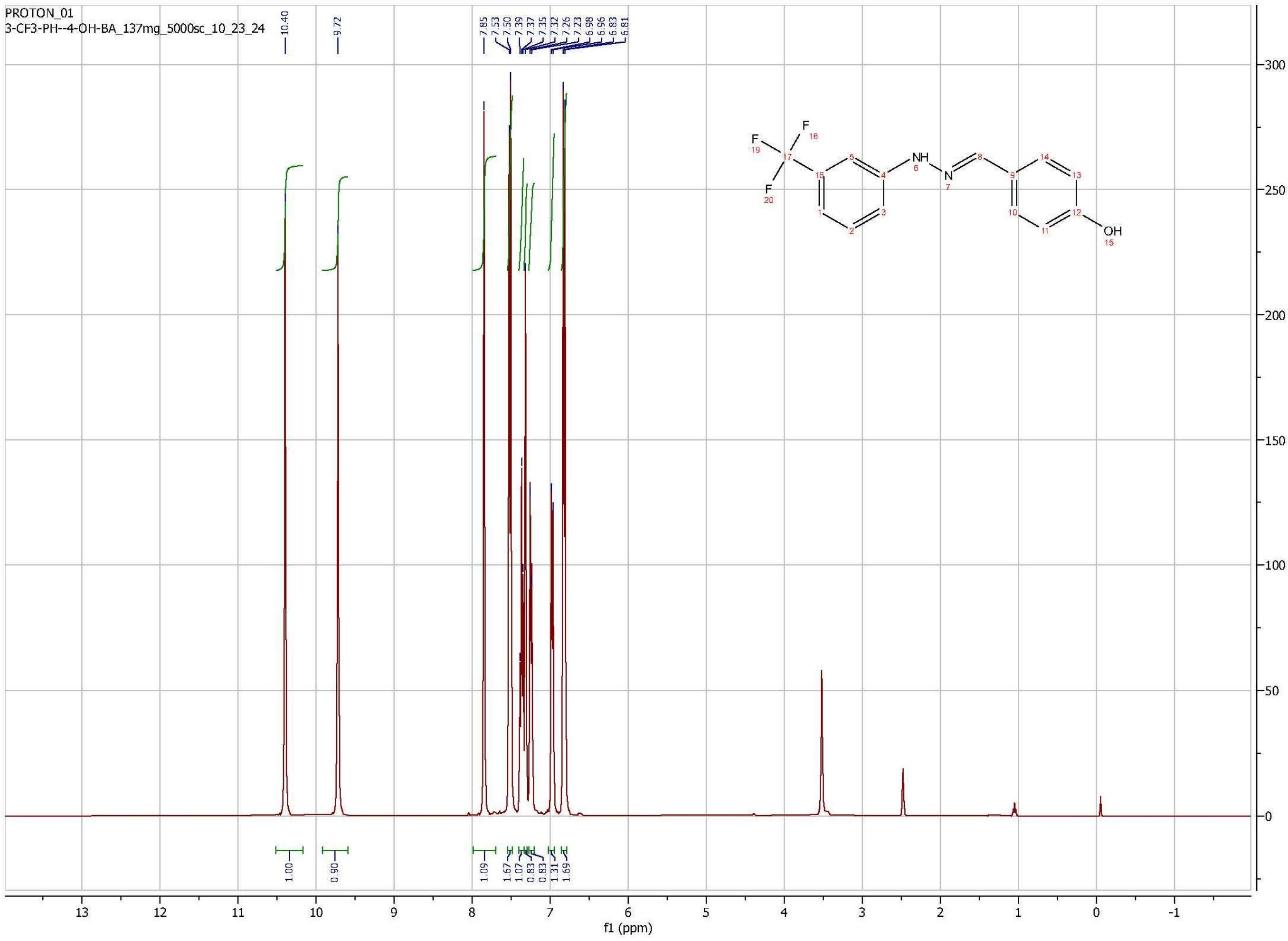

Fig. S25. ^{1}H NMR spectrum of (*E*)-1-(4-hydroxybenzylidene)-2-(3-(trifluoromethyl)-phenylhydrazine (**7**).

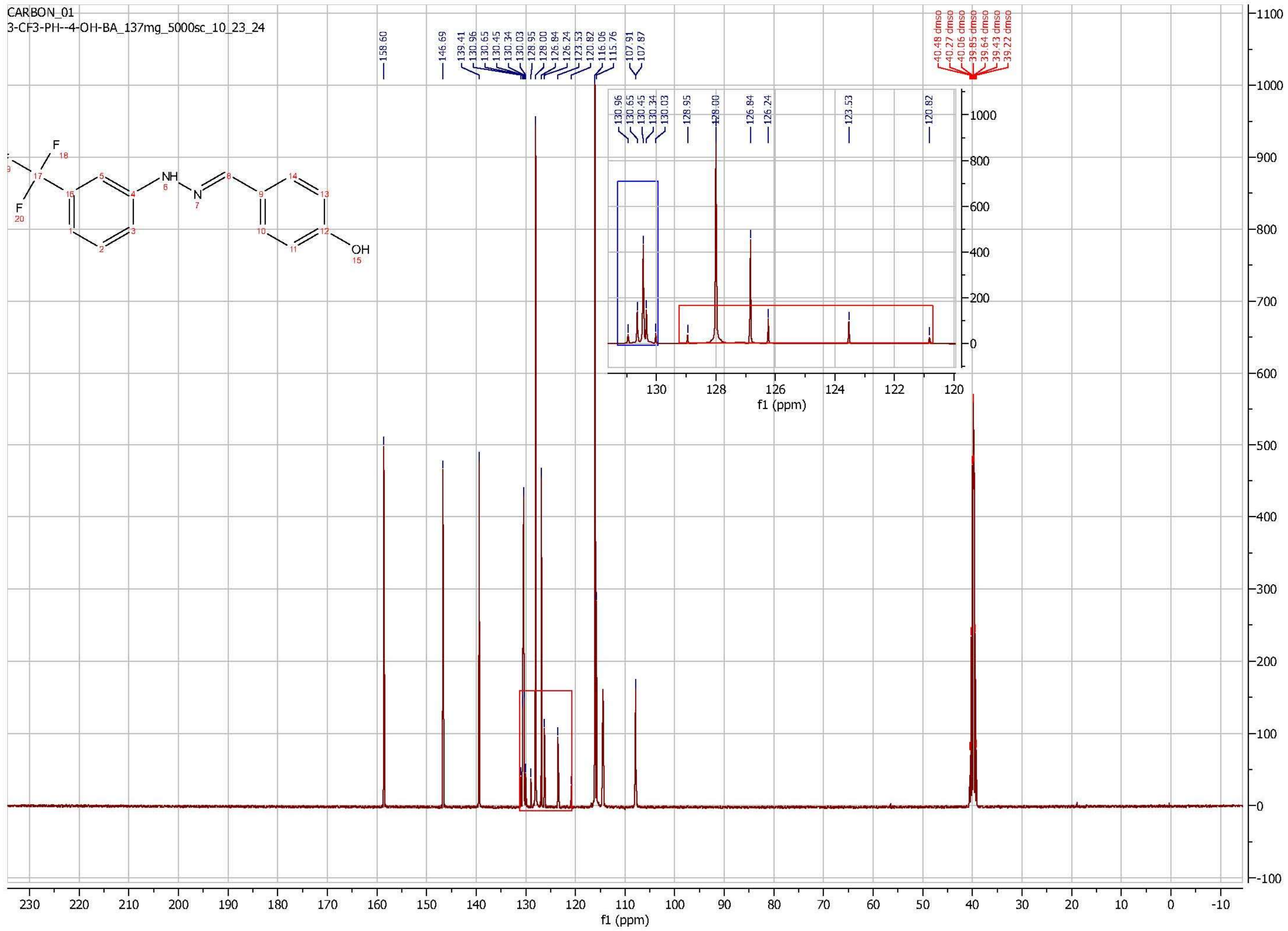

Fig. S26. ^{13}C NMR spectrum of (*E*)-1-(4-hydroxybenzylidene)-2-(3-(trifluoromethyl)-phenylhydrazine (**7**).

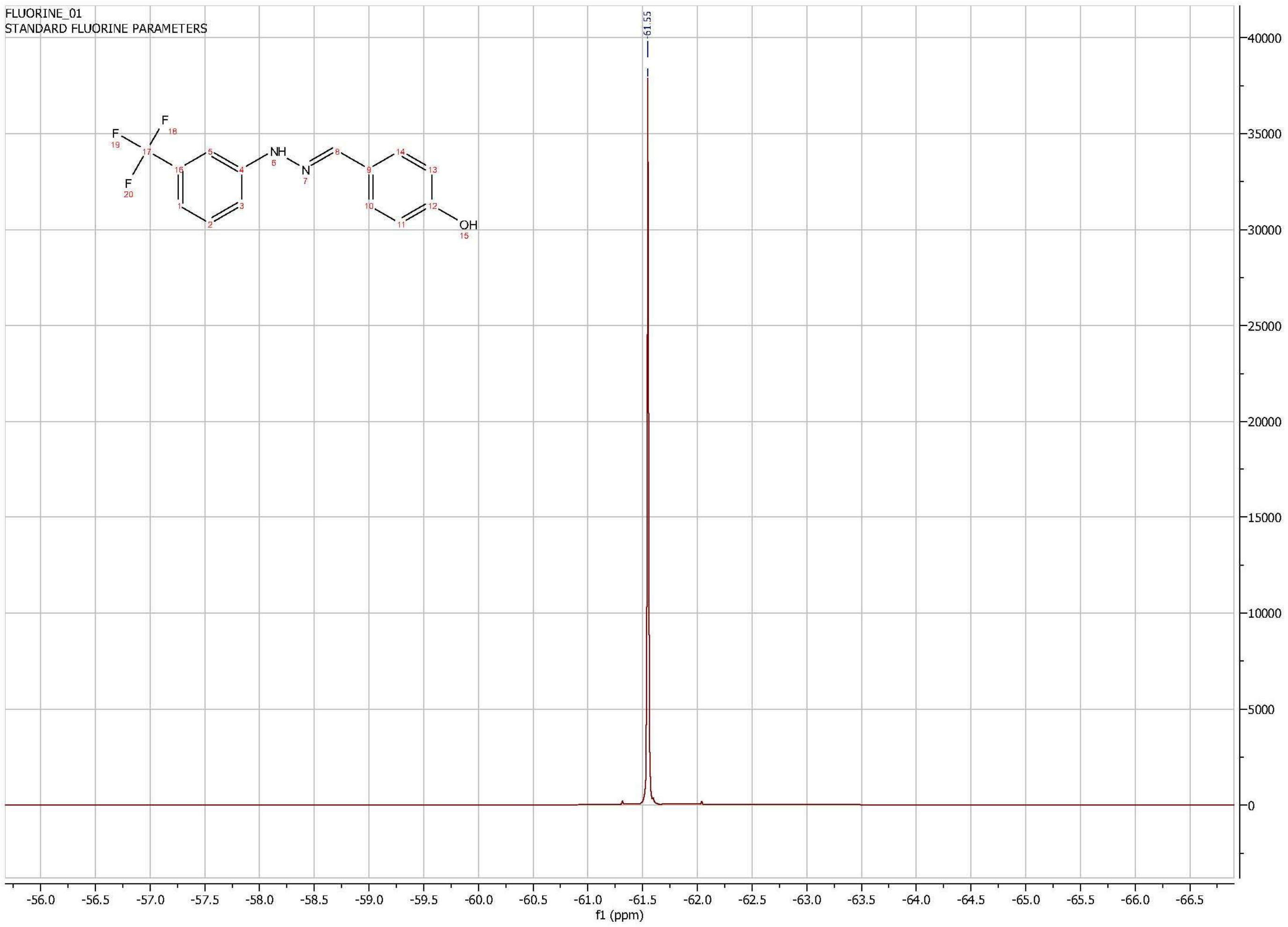

Fig. S27. ^{19}F NMR spectrum of (*E*)-1-(4-hydroxybenzylidene)-2-(3-(trifluoromethyl)-phenylhydrazine (**7**).

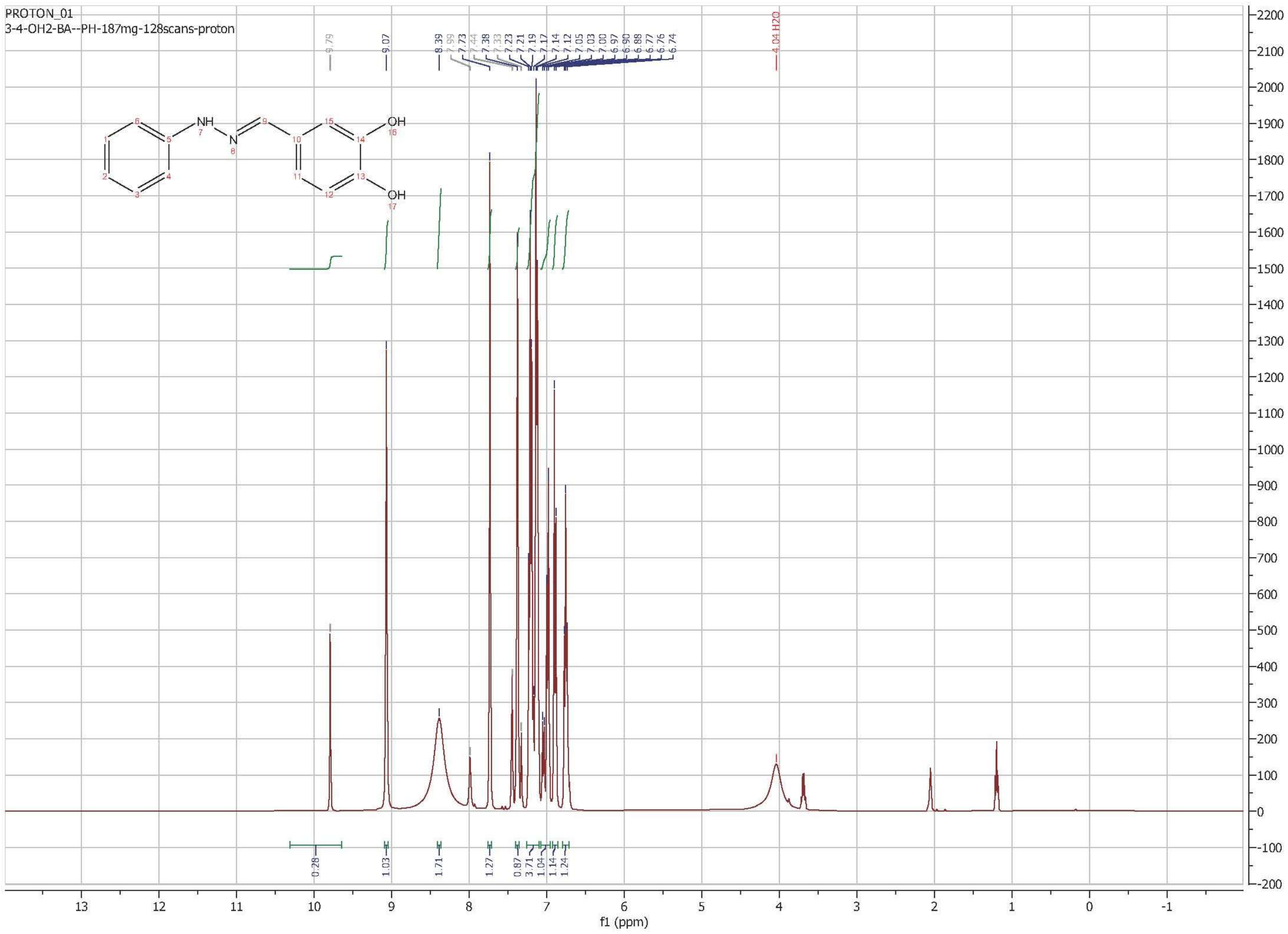

Fig. S28. ^{1}H NMR spectrum of (*E*)-1-(3,4-dihydroxybenzylidene)-2-phenylhydrazine (**8**).

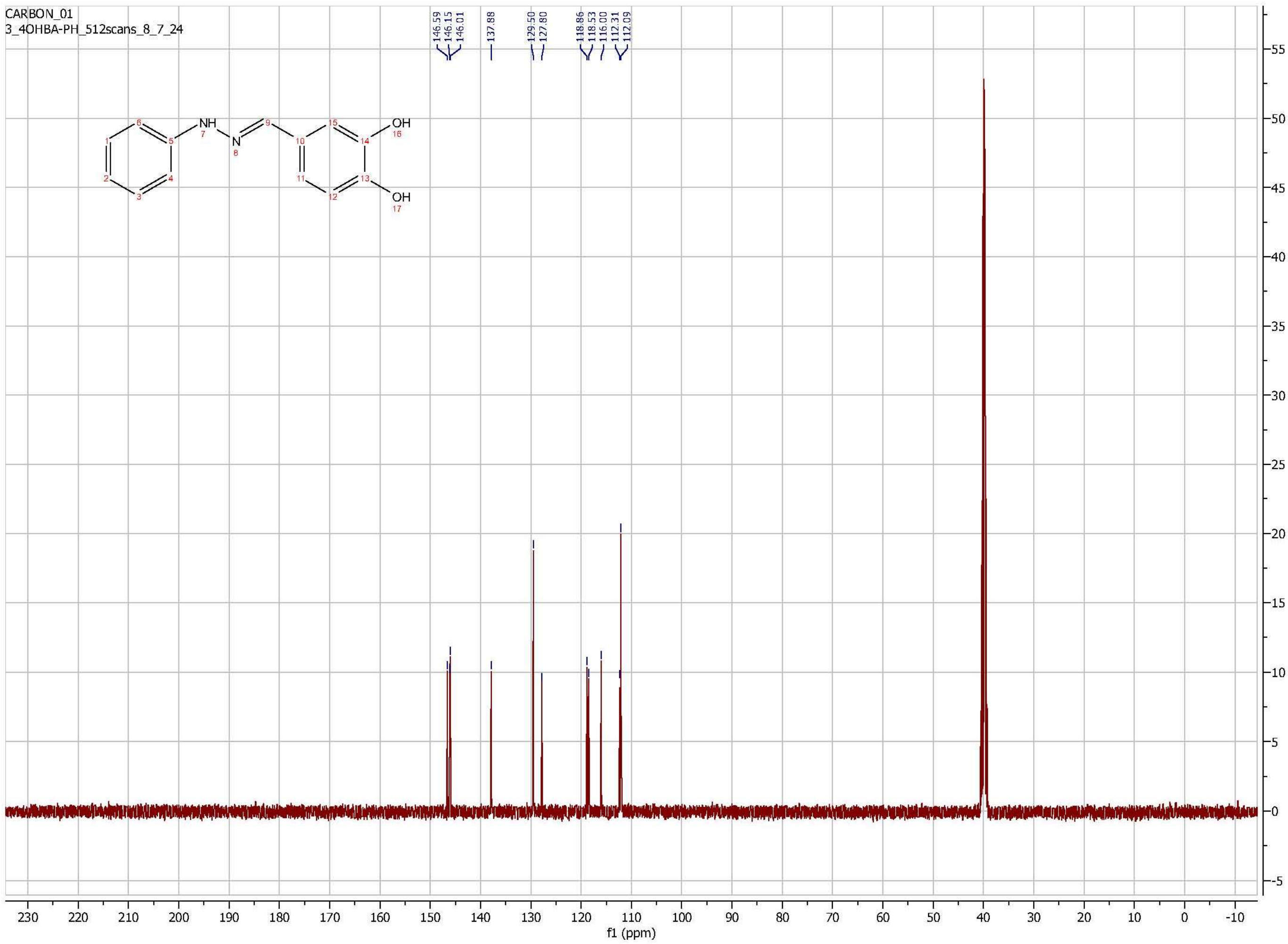

Fig. S29. ^{13}C NMR spectrum of (*E*)-1-(3,4-dihydroxybenzylidene)-2-phenylhydrazine (**8**).

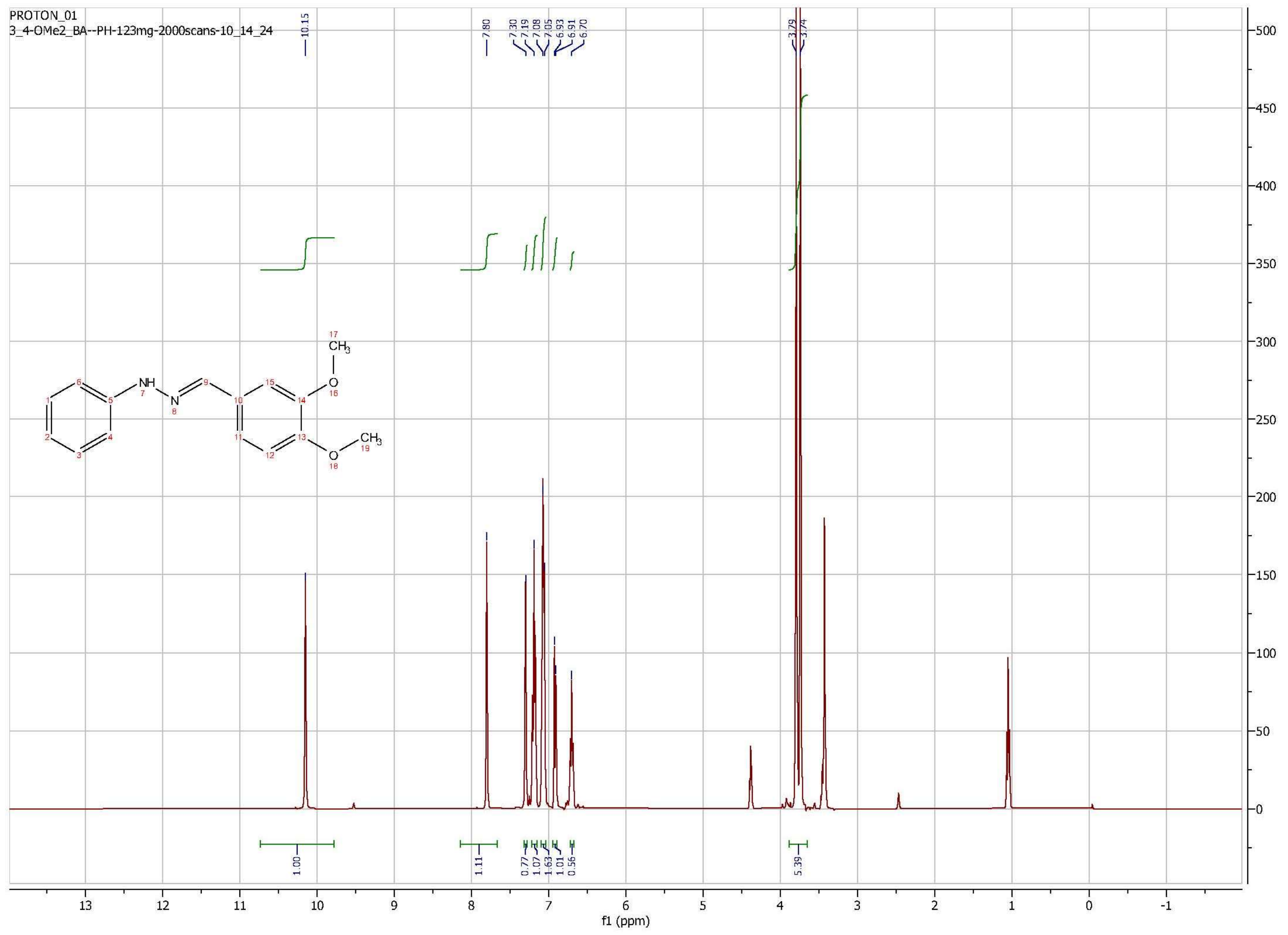

Fig. S30. ^{1}H NMR spectrum of (*E*)-1-(3,4-dimethoxybenzylidene)-2-phenylhydrazine (**9**).

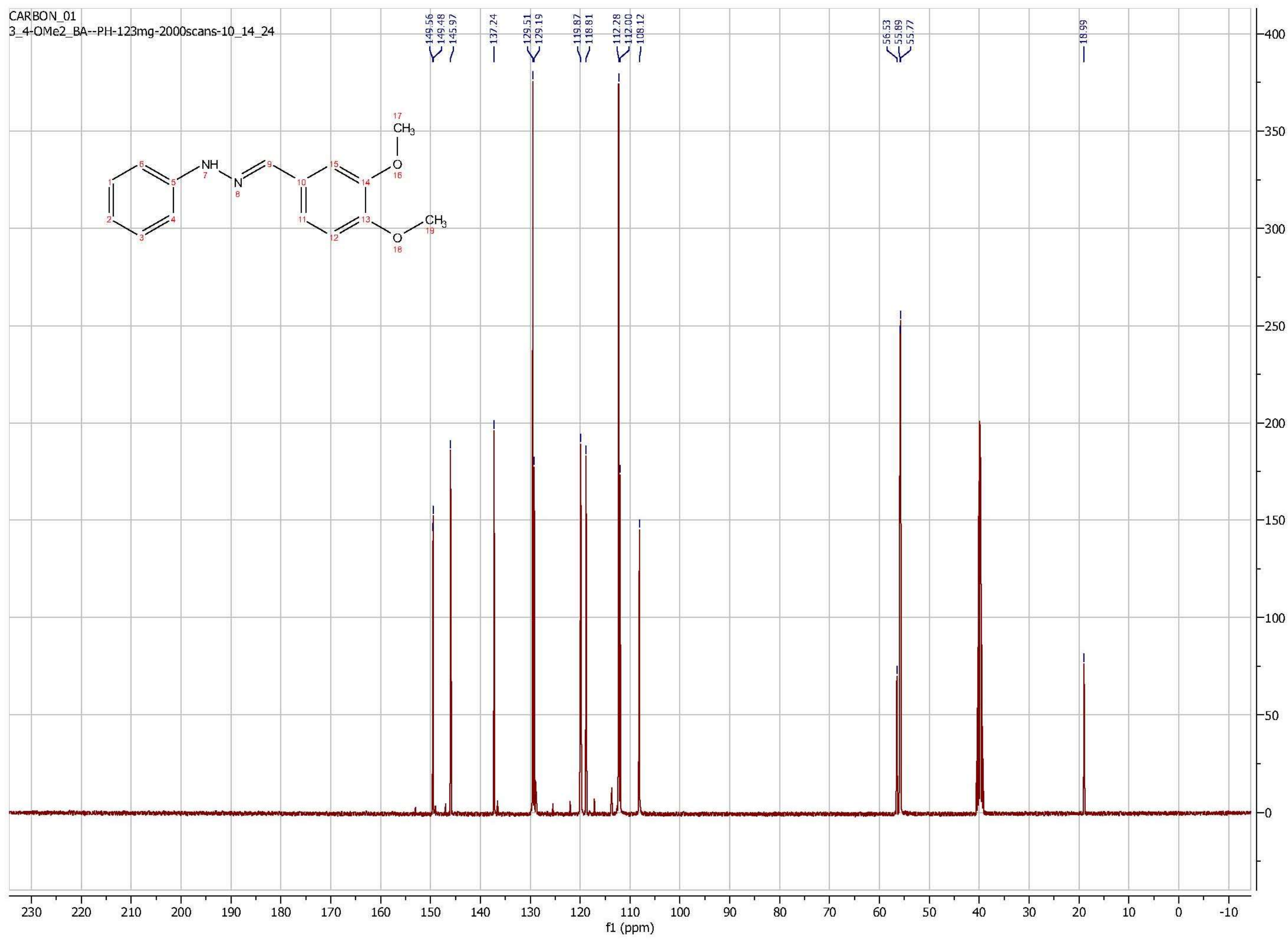

Fig. S31. ^{13}C NMR spectrum of (*E*)-1-(3,4-dimethoxybenzylidene)-2-phenylhydrazine (**9**).
